# Genome-wide chromatin profiling reveals a nonlimiting role for RXR in macrophage-like cells stimulated with multiple nuclear receptor agonists

**DOI:** 10.1016/j.jbc.2026.111486

**Published:** 2026-04-22

**Authors:** Hamidreza Mianesaz, Loránd Göczi, Dóra Bojcsuk, Safoura Ghalamkari, Lina Fadel, Szilárd Póliska, András Penyige, László Nagy, Gergely Nagy, György Vámosi, Lajos Széles

**Affiliations:** 1 Department of Medical Genetics, Faculty of Medicine, University of Debrecen, Debrecen, Hungary; 2 Doctoral School of Molecular Cellular and Immune Biology, University of Debrecen, Debrecen, Hungary; 3 Department of Biochemistry and Molecular Biology, Faculty of Medicine, University of Debrecen, Debrecen, Hungary; 4 Institute for Diabetes and Endocrinology IDE, Neuherberg, Germany; 5 Department of Medicine and Biological Chemistry, Johns Hopkins University School of Medicine, Institute for Fundamental Biomedical Research, Johns Hopkins All Children's Hospital, Saint Petersburg, Florida, USA; 6 Department of Biophysics and Cell Biology, Faculty of Medicine, Doctoral School of Molecular Medicine, University of Debrecen, Debrecen, Hungary

**Keywords:** nuclear receptor, macrophage, ChIP-sequencing (ChIP-seq), transcriptomics, transcription factor, retinoid X receptor (RXR), vitamin D receptor (VDR)

## Abstract

Retinoid X receptor (RXR) is an obligate heterodimerization partner for many nuclear receptors. In the absence of ligands, RXR occupies thousands of genomic regions, with its binding landscape predominantly determined by cell identity. In the presence of agonists of RXR or its partners, RXR occupancy is changed at a subset of binding regions. The characteristics of these ligand-responsive binding regions remain largely unexplored. We used ChIP-seq to profile RXR occupancy in PMA-differentiated THP-1 cells treated with agonists of RXR or partner receptors, including RARα, VDR, PPARδ, PPARγ, LXRs, and TR, or a “cocktail” containing multiple agonists. The RXR agonist LG268 produced a stronger increase in RXR occupancy than any of the six partner-receptor agonists or their combination. The relevance of ligand-induced RXR peaks was confirmed by the analyses of motif enrichment and RXR occupancy at regulatory elements of target genes. RXR binding was investigated in more detail in cells treated with the VDR agonist, calcitriol. Calcitriol markedly enhanced VDR binding, but the corresponding increase in RXR occupancy was less pronounced. We found that both ligand-induced and unresponsive RXR peaks were involved in gene regulation, and only a small subset (∼3%) of calcitriol-regulated genes exhibited decreases in both RXR binding and mRNA levels in response to combined agonist treatment. These results support a model in which RXR functions as a nonlimiting module in a macrophage-like cell type, and interference between pathways is minimally attributable to RXR sequestration.

Retinoid X receptors (RXRs) belong to the nuclear receptor (NR) superfamily and exist as three isotypes, RXRα, RXRβ, and RXRγ, with distinct tissue distributions (1, 2, 3, 4). RXRs are obligate dimerization partners for multiple NRs, including vitamin D receptor (VDR), thyroid hormone receptors (TRs), retinoic acid receptors (RARs), liver X receptors (LXRs), and peroxisome proliferator-activated receptors (PPARs) (3, 4, 5). RXRs can also form homodimers, although the specific functional role of these homodimers is not completely elucidated (2, 4, 6, 7, 8, 9). RXRs can be activated by endogenous ligands, such as 9-cis-retinoic acid, 9-cis-13,14-dihydroretinoic acid, and docosahexaenoic acid, and by synthetic agonists (2, 10, 11, 12). High-affinity endogenous ligands have been identified for approximately two-thirds of the 19 RXR partners in humans (13, 14, 15). A subset of RXR heterodimers can be activated by RXR ligand in the absence of ligands of the RXR partner. These so-called “permissive heterodimers” include LXR-RXR, PPAR-RXR, FXR-RXR, and PXR-RXR (2, 3, 4).

Transcriptional responses induced by the ligands of RXR and its partners are mediated by DNA-bound receptors, although nongenomic actions have also been described. Early studies in the NR field (1980s-1990s), which relied on *in vitro* assays such as DNase I footprinting and EMSAs, provided foundational insights into how RXR-containing heterodimers recognize DNA motifs (3, 16, 17). These studies revealed that RXR homodimers and heterodimers bind response elements (REs) composed of direct repeats of the core AGGTCA motif, separated by defined nucleotide spacers ranging from 0 to 5 bases (3, 4, 6, 16, 18). Because different RXR heterodimers exhibit distinct sequence preferences, they regulate distinct (although partly overlapping) sets of target genes. The DNA-binding behavior of RXR heterodimers differs markedly from the DNA-binding of the glucocorticoid receptor (GR) and other steroid receptors. In the absence of a ligand, the GR is predominantly inactive and localized to the cytoplasm; after ligand binding, the GR translocates to the nucleus, occupies its response elements, and induces transcription of its target genes (14, 19, 20). In contrast, the activity of RXR heterodimers can be described as a ligand-induced molecular switch. In the absence of agonists, RXR heterodimers bind DNA and recruit corepressors such as SMRT and N-CoR. In the presence of agonists, they are associated with coactivators, including p300 and other histone acetyltransferases, as well as the Mediator complex, thereby activating gene expression. This ligand-induced molecular switch has been documented for multiple RXR partners, including RAR, TR, and VDR (21, 22, 23).

Advances in live-cell imaging and single-molecule tracking revealed that receptor–chromatin interactions are highly dynamic. Receptor–chromatin interactions initially characterized for GR indicated that individual molecules bind transiently but achieve functional regulation at the population level (24). We and others also observed this dynamic aspect of DNA binding in the case of other NRs, RXR, and its heterodimerization partners, including RAR, VDR, and PPARγ, using live-cell imaging techniques (25, 26, 27, 28, 29, 30, 31, 32).

Methods of functional genomics, including chromatin immunoprecipitation coupled with high-throughput sequencing (ChIP-seq), assay for transposase-accessible chromatin using sequencing, and RNA sequencing (RNA-seq), have enabled mapping of NR binding across the genome, assessment of chromatin accessibility, occupied DNA motifs, and integration with transcriptional outputs (33, 34). Insights from these studies have shown that a large proportion of binding regions of RXR heterodimers do not contain canonical response elements, and binding is largely determined by local chromatin accessibility and other transcription factors (TFs). Regarding RXR, previous ChIP-seq studies have shown how cellular differentiation and exposure to various ligands shape RXR binding landscapes (35, 36, 37, 38, 39, 40). Notably, RXR binding landscapes are more strongly influenced by cell identity than by ligands of RXR or its partners (35, 38).

In this study, we investigated the RXR chromatin binding in differentiated THP-1 cells. THP-1 is a human monocytic leukemia cell line widely used as a model to study monocyte and macrophage biology (41). Differentiation of THP-1 cells can be induced by phorbol-12-myristate-13-acetate (PMA) and other compounds, including LPS, IFN-γ, IL-4, and TGF-β (42, 43, 44), resulting in a macrophage-like phenotype that is suitable for investigating widely expressed NRs, including RXR and its heterodimeric partners (45, 46, 47, 48, 49, 50, 51). Using PMA-differentiated THP-1 (PMA-THP-1) cells, our aim was to determine the extent and patterns by which ligands of RXR or its heterodimeric partners reshape the RXR binding landscape and to examine whether RXR functions as a limiting module when multiple ligands are present together. The latter issue is particularly intriguing because crosstalk between NRs, including RXR heterodimers, has been well documented (5, 52, 53). One potential mechanism at the DNA level is that the common dimerization partner is redistributed or sequestered upon binding of a second agonist (52). While studies using *in vitro* and reporter gene assays, and our previous live-cell imaging experiments have suggested that limited RXR availability may contribute to such interference (27, 29, 54, 55, 56, 57, 58), this mechanism has not yet been investigated in the context of chromatin binding using ChIP-seq.

ChIP-seq provides not only a genome-wide catalog of NR-binding sites but also information on the binding intensity of the investigated factors (59). The ChIP-seq method has limitations, as it provides a population-averaged, static snapshot. Moreover, a single ChIP-seq peak may contain multiple binding sites for a given TF, and comparison of different TFs binding events or different antibodies are challenging using ChIP-seq (60). Despite these limitations, ChIP-seq provides a reproducible readout of relative TF occupancy across the genome and can be considered semiquantitative. Differences in peak size (occupancy signal) within one sample are not random, and ChIP-seq peak size has biological meaning. This is evidenced by a series of observations, including consistent rankings across biological replicates and correlations with independent biological features such as DNA motif, cofactor recruitment, and histone modifications (61). Transcriptional output also correlates with peak size, with highly expressed genes often associated with large peaks and/or multiple TF binding regions (62). These features collectively support that the ChIP-seq peak sizes reflect how RXR binding is affected under various conditions. An increase or decrease in ChIP-seq peak size may indicate that the RXR binding is increased or decreased, respectively.

In this study, systematic analysis of RXR binding patterns revealed that RXR functions as a nonlimiting, shared component in transcriptional activation by its partners in PMA-THP-1 macrophage-like cells, although this role may vary in other cell types with differing RXR and partner expression patterns.

## Results

### ChIP-seq meta-analysis places RXR among the NRs least influenced by ligand treatment with respect to overall genomic occupancy

**Figure 1:**
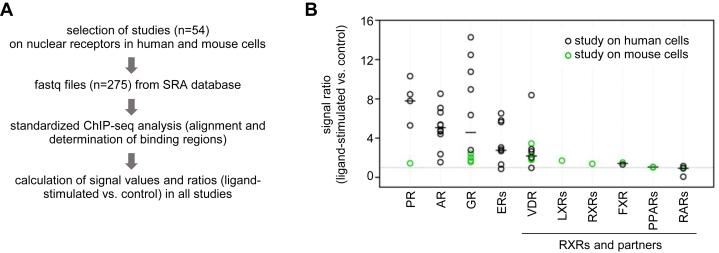
**ChIP-seq meta-analysis of ligand effects on DNA occupancy by nuclear receptors**. *A*, schematic outline of the workflow for the meta-analysis of public ChIP-seq datasets. *B*, graph showing signal ratios obtained from the ChIP-seq meta-analysis. Each dot represents a dataset from an individual study. Signal values were calculated at binding regions identified in each dataset, and the ratio of ligand-stimulated to control signals was determined. The control samples were either vehicle-treated or unstimulated. The median of ratio values for each nuclear receptor is indicated when data from more than one study were available. UniProt symbols are shown, except for PR (PRGR), AR (ANDR), GR (GCR), ERs, (ESR1, ESR2), LXRs (NR1H3, NR1H2), and FXR (NR1H4).

ChIP-seq datasets from public databases were used to assess the ligand-induced changes in DNA binding of RXR and other NRs in various cell types (Table S1). Relevant ChIP-seq studies on NRs were selected based on several criteria (see Experimental procedures). For example, only studies that included both control (untreated or vehicle) and ligand-stimulated conditions were selected. Although all 27 ligand-responsive NRs were surveyed (13, 14, 15, 63) (Table S2), many NRs did not have ChIP-seq datasets or the datasets did not pass all selection criteria (Tables S1). The selected datasets (27, 38, 39, 40, 50, 64, 65, 66, 67, 68, 69, 70, 71, 72, 73, 74, 75, 76, 77, 78, 79, 80, 81, 82, 83, 84, 85, 86, 87, 88, 89, 90, 91, 92, 93, 94, 95, 96, 97, 98, 99, 100, 101, 102, 103, 104, 105, 106, 107, 108, 109, 110, 111, 112, 113) were downloaded and processed using a standardized analysis pipeline (Fig. 1*A*). The ratio of occupancy in ligand-stimulated samples compared to control samples was calculated for each study. These ratios, which provide a single value per study, are summarized in Figure 1*B* and Table S1. Consistent with previous research, steroid receptors, including GRs and androgen receptor, exhibited pronounced ligand-induced changes in overall genomic occupancy, resulting in minimal correlation observed between ChIP-seq datasets of control and ligand-treated samples (Fig. 1*B* and Fig. S1, *A*–*B*). Significant differences in ratios were observed among studies on the same receptor, most likely due to the level of endogenous ligands in various cell types, the experimental setup, and technical issues. The number of datasets available for RXR and its partners was lower than the number of datasets for steroid receptors. Among RXR and its partners, only modest ligand-induced changes in overall genomic occupancy were detected. The VDR exhibited the highest degree of ligand-induced changes, although these changes were still lower than the changes observed for steroid receptors.

### Overall genomic occupancy of RXR is largely unchanged following ligand stimulation in PMA-THP-1 cells

**Figure 2:**
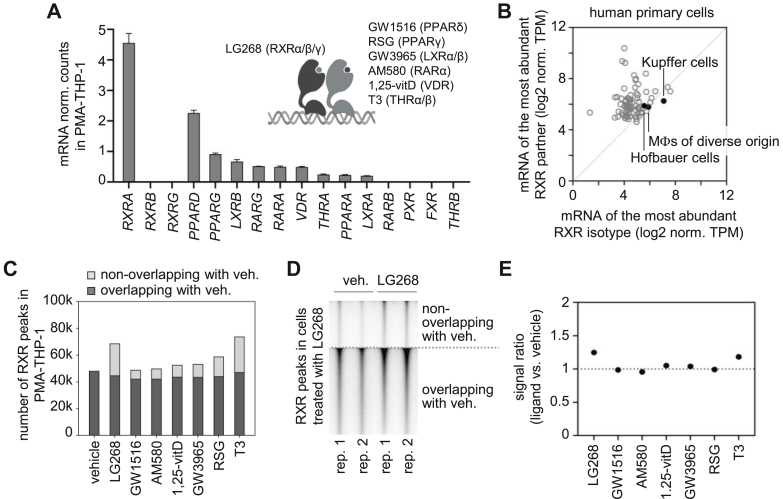
**Comparison of RXR binding regions in PMA-THP-1 cells treated with vehicle or ligands for RXR and its partners**. *A*, expression of RXRs and their dimerization partners in PMA-differentiated THP-1 (PMA-THP-1) cells, as determined by RNA-seq. The mean and standard deviation of the three replicates of vehicle-treated cells are shown. The inset lists the ligands used in subsequent experiments. HGNC-approved gene symbols are shown, except for *LXRA* (*NR1H3*), *LXRB* (*NR1H2*), *PXR* (*NR1I2*), and *FXR* (*NR1H4*). *B*, expression levels of RXR and its most highly expressed partner in various primary cell types. RNA-seq data were obtained from the Human Protein Atlas. The *blackdots* represent primary human macrophages. *C*, the total number of reproducible RXR peaks determined in vehicle- and ligand-stimulated PMA-THP-1 cells, color-coded for peaks detected in both vehicle (veh) and ligand-stimulated cells or exclusively in ligand-stimulated cells. PMA-THP-1 cells were treated with vehicle or agonists for 2 h, and RXR-binding regions were determined by ChIP-seq in two biological replicates. The reproducible RXR peaks (peaks detected in both replicates of each condition) were retained using intersectBed and merged using mergeBed (bedtools). *D*, read distribution plot showing RXR ChIP-seq signals in 2 kb windows. RXR peaks identified in LG268-treated cells are used for this analysis and the signals in two replicates of vehicle- and LG268-treated cells are displayed. *E*, signal ratios obtained from RXR ChIP-seq analysis. Signals were calculated at binding regions identified under each condition, and the ratio of ligand-stimulated to control signal was computed. 1,25-vitD, 1α,25-dihydroxyvitamin D3; GW1516, GW501516; HGNC, HUGO Gene Nomenclature Committee; LG268, LG100268; RSG, rosiglitazone; T3, triiodothyronine.

Using PMA-THP-1 as a cellular model, the effects of ligands activating RXR and its heterodimerization partners on RXR chromatin binding were investigated. Using our previous RNA-seq data (64), ligand-inducible RXR dimerization partners that are highly expressed in PMA-THP-1 cells were identified (Fig. 2*A*). Of note, based on protein abundance, the rank of these receptors could be different. *RXRA* was the most abundant RXR isotype at the mRNA level, whereas *RXRB* and *RXRG* were expressed at very low levels. The RXR partners expressed at high or intermediate levels included *PPARD*, *PPARG*, *LXRB*, *RARG*, *RARA*, *VDR*, *THRA*, *PPARA*, and *LXRA*. Notably, *RXRA* mRNA expression was higher than the expression of its partners in PMA-THP-1 cells (Fig. 2*A*). Analysis of Human Protein Atlas mRNA datasets (114) revealed that expression of the most abundant RXR isotype was higher than or comparable to that of its partners in many primary human cell types, including macrophages (Fig. 2*B*). Nevertheless, in many other cell types, the expression of RXR partners substantially exceeded that of the most abundant RXR isotype (Fig. 2*B* and Fig. S2*A*).

For the ChIP-seq analysis, LG268, an RXR agonist, and six ligands activating highly expressed RXR partners were selected (Fig. 2*A*). PMA-THP-1 cells were stimulated with the selected ligands at concentrations determined in pilot experiments and previous studies (7, 115, 116) to achieve maximal receptor activation. RXR-binding regions were identified in all samples using ChIP-seq, and reproducible RXR peaks (peaks detected in both replicates of each condition) were defined (Fig. 2*C*). The number of reproducible RXR peaks in the ligand-treated cells overlapping with those in vehicle-treated cells was also determined (Fig. 2*C*). Notably, most binding regions detected in ligand-treated samples but absent in vehicle-treated samples exhibited relatively low RXR signal intensity (Fig. 2*D* and Fig. S2*B*). We found that RXR binding signal ratios (ligand-stimulated *versus* control) were usually close to 1.0; the highest values were observed for LG268 and T3 (1.24 and 1.18, respectively) (Fig. 2*E*). These results indicate that overall RXR occupancy is largely unchanged in PMA-THP-1 cells, irrespective of the used ligand, consistent with previous observations in mouse bone marrow–derived macrophages (38).

### Identification of RXR peaks with changes in the occupancy upon ligand treatment

Although signal ratios did not indicate robust changes in overall RXR occupancy, this did not exclude the possibility of RXR redistribution at specific genomic loci. To identify the RXR peaks showing induction or reduction after ligand treatment in PMA-THP-1 cells, the DiffBind package was used, applying fold change (FC) and FDR cutoffs. Among the tested ligands, LG268 had the strongest effect, yielding the highest number of ligand-responsive RXR peaks with 8820 induced (FC > 1.5, adjusted *p* < 0.05) and 10,430 reduced (FC < 0.66, adjusted *p* < 0.05) RXR peaks. Ligands of RXR partners had more modest effects. Typically, fewer than a thousand RXR peaks were significantly induced upon ligand treatment, while few or no peaks were significantly reduced by these ligands (Fig. S3*A*).

These results prompted us to consider filtering based solely on FC using 1.5 and 0.66 cutoffs, as an alternative peak selection strategy for downstream analyses. As expected, using “FC-only” approach, a considerably higher number of ligand-responsive RXR peaks were identified (Fig. S3*B*). Despite the apparent limitation of the FC-only approach (namely, the inclusion of peaks with greater variability between replicates), it also offers certain advantages. Analysis of larger peak sets enables identifying global patterns, motifs, and correlations, providing a more comprehensive view of RXR redistribution. Moreover, FC-only approach may allow a more balanced classification of peaks into “ligand-responsive” and “ligand-unresponsive” categories. Although applying stringent statistical thresholds reduces putative false positives in the “ligand-induced” and “ligand-reduced” groups, this comes at the cost of classifying many moderately changing peaks that fail to pass the statistical thresholds as ligand-unresponsive. In this context, FC-only approach can be considered as a practical compromise.

To assess the biological relevance of peaks identified using the FC-only approach, we performed three analyses on RXR peaks induced by 1α,25-dihydroxyvitamin D3 (1,25-vitD, also known as calcitriol) treatment (n = 2390; Fig. S3*B*) as a case study. This set was divided into two subsets for the three analyses: RXR peaks passing the FDR cutoff (“FC-pass and significant”; FC > 1.5 and FDR < 0.05) and RXR peaks failing to pass the FDR cutoff (“FC-pass and non-significant”; FC > 1.5 and FDR ≥ 0.05) (Fig. S3*C*). First, we evaluated the enrichment of DR3 motifs, the canonical response element for VDR-RXR. We found that DR3 was enriched not only in the "FC-pass and significant" peak set (72%) but also in the "FC-pass and non-significant" set (30%; Fig. S3*D*), suggesting that many regions in both subsets serve as VDR–RXR binding sites. Second, we examined RXR signal intensities in the two subsets across biological replicates (Fig. S3, *E*–*F*). RXR occupancy exhibited consistent ligand-dependent increases in both subsets across replicates. Notably, "FC-pass and non-significant" peaks generally exhibited lower signals and smaller FCs than "FC-pass and significant" peaks (Fig. S3*F*). Third, we assessed the correlation between the occupancy signals in the two replicates for vehicle- and 1,25-vitD–treated samples within each subset. While the replicates of each condition generally showed a high correlation in both subsets, signals for "FC-pass and non-significant" peaks exhibited greater variability across replicates compared with "FC-pass and significant" peaks (Fig. S3*G*). These results suggest that many RXR peaks with smaller FCs and greater variability between replicates fail to pass the FDR cutoff yet may still be biologically relevant.

**Figure 3:**
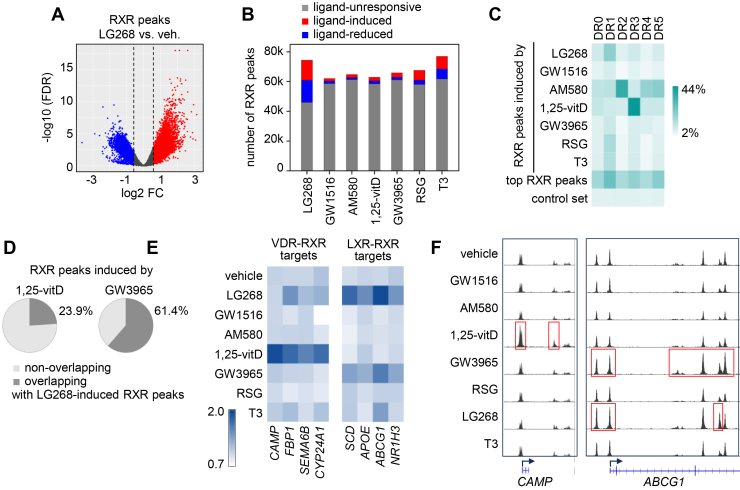
**Differential RXR binding in response to ligand treatment**. *A*, comparison of RXR peaks in LG268-treated and vehicle-treated PMA-THP-1 cells, showing log2 fold changes (FCs) in read counts (x-axis) and statistical significance (-log 10 *p*-value, y-axis). Peaks with FC > 1.5 or < 0.66 relative to vehicle are color-coded in *red* and *blue*, respectively. *B*, number of ligand-unresponsive, ligand-induced (FC > 1.5), and ligand-reduced (FC < 0.66) exploratory RXR peak sets. *C*, frequency of DNA motifs in various ligand-induced exploratory RXR peak sets, in the *top* RXR peaks, and in a control set of randomly selected, size-matched sequences (n = 5000). The RXR peaks were ranked based on the occupancy values in vehicle-treated cells, and the top 5′000 peaks were considered as “*top* RXR peaks.” *D*, proportion of exploratory RXR peak sets induced by 1,25-vitD or GW3965 that overlap with the LG268-induced exploratory RXR peak set. *E*, heatmap showing normalized RXR occupancy signals within TSS ± 25 kb of selected target genes of VDR and LXR. Values were normalized to the median. *F*, Integrative Genomics Viewer snapshot of RXR ChIP-seq signals at representative target genes of VDR-RXR (*CAMP*) and LXR-RXR (*ABCG1*). Signal tracks are shown for different treatment conditions, with track scales set to 0 to 60 for *CAMP* and 0 to 50 for *ABCG1*. Regions within *redsquares* are induced by the indicated ligand (FC > 1.5). DR, direct repeat; TSS, transcription start site.

Based on our results, the FC-only approach was used to define the ligand-responsive RXR sets for subsequent analyses. To indicate that no stringent statistical filtering was applied, we refer to them as ligand-responsive “exploratory RXR sets” (Fig. 3, *A* and *B*). The number of peaks in each set, along with their corresponding FC and FDR values, is provided in Table S3.

### Characterization of ligand-responsive exploratory RXR peak sets: motif enrichment, overlap, and association with target genes

We performed a series of downstream analyses to characterize the exploratory ligand-responsive RXR peak sets. DNA motif enrichment was analyzed in ligand-induced and ligand-reduced RXR peaks, as well as in the top RXR peaks and the control set (Fig. 3*C* and Fig. S4*A*). Motif analysis revealed that all canonical DR0-DR5 motifs were enriched in the top RXR peak set from vehicle-treated cells, while in the ligand-induced RXR peaks, they usually reflected the binding preferences of the heterodimers (3, 4, 16, 18) (Fig. S4*B*). For example, 1,25-vitD–induced peaks were enriched for DR3 motifs, whereas RARα ligand-induced peaks (AM580) were enriched for DR1, DR2, and DR5 motifs, consistent with the motif preferences of VDR-RXR and RAR-RXR, respectively. The enrichment of DR4 motifs was also detected in the RARα ligand-induced set, contrary to the results from previous studies of RAR-RXR DNA motif preferences. DR4 motifs were lowly enriched in GW3965-induced and T3-induced RXR peak sets, despite the known DR4 preference of LXR-RXR and TR-RXR heterodimers (Fig. 3*C*). The reason for the low enrichment is unclear. Notably, DNA motif enrichment analysis of ligand-reduced RXR peaks revealed that these regions were often enriched for motifs other than the canonical motif of the corresponding liganded receptor (Fig. S4*A*), suggesting that RXR is recruited to the regions occupied by activated RXR-dimer and withdrawn from regions occupied by other RXR dimers. The observed redistribution is consistent with our previous findings in HEK293 cells, where nuclear translocation assays and fluorescence correlation spectroscopy demonstrated that, upon agonist treatment, the liganded NR consistently dominated in heterodimerization with RXR (27). Nevertheless, although motif analysis suggests that the redistribution is ligand-specific, it affects only a small proportion of RXR peaks.

We further characterized the ligand-induced RXR peaks by assessing the overlap between RXR peak sets induced by LG268 and ligands of RXR partners (Fig. 3*D* and Fig. S4*C*). The pattern of overlap generally mirrored the permissive or nonpermissive nature of RXR heterodimers. For example, 1,25-vitD–induced RXR peaks showed the lowest overlap with LG268-induced peaks (23.9%), consistent with VDR-RXR heterodimers being nonpermissive to RXR ligands. In contrast, RXR peaks induced by agonists for NRs forming permissive heterodimers, LXR-RXR, PPARδ-RXR, and PPARγ-RXR, displayed a high degree of overlap with LG268-induced RXR peaks (61.4%, 54.1%, and 63.5%, respectively). Unexpectedly, T3-induced RXR peaks also showed a high overlap with LG268-induced peaks (Fig. S4*C*). Ligand-induced RXR peaks were frequently close to known target genes for RXR partners. For example, VDR and LXR target genes were often associated with 1,25-vitD– and GW3965-induced RXR peaks, respectively (Fig. 3, *E* and *F*). Notably, RXR binding was also enhanced at regions associated with LXR target genes in response to LG268, reflecting the permissive nature of LXR-RXR heterodimers.

### 1,25-vitD–responsive genes are associated with regulatory regions at which RXR binding is either induced or unchanged by treatment with 1,25-vitD

We aimed to characterize RXR-binding regions with respect to their gene regulatory activity and other genomic features. For this characterization, we focused on genes regulated by the VDR agonist 1,25-vitD and mapped and characterized RXR binding regions that potentially contribute to their regulation. We chose 1,25-vitD for this analysis for two reasons: (i) the binding landscape of its receptor, VDR, has already been characterized in PMA-THP-1 cells by ChIP-seq in our previous study (64) and was used in this analysis, and (ii) VDR activation by 1,25-vitD induced the largest changes in overall RXR occupancy among all RXR partners analyzed (Fig. 1*B*). To implement this investigation, RNA-seq experiments were performed in cells treated with vehicle or 1,25-vitD for 6 h. New RXR ChIP-seq experiments were also performed using 1-h vehicle or 1,25-vitD treatments, matching the treatment time point of the previously generated VDR ChIP-seq dataset. To evaluate the regulatory activity of RXR binding regions, we analyzed MED1 ChIP-seq datasets generated in our previous work from cells treated with vehicle or 1,25-vitD for 2 h (64) and performed novel Histone H3 acetylated at lysine 27 (H3K27ac) ChIP-seq experiments in cells treated with vehicle or 1,25-vitD for 6 h.

**Figure 4:**
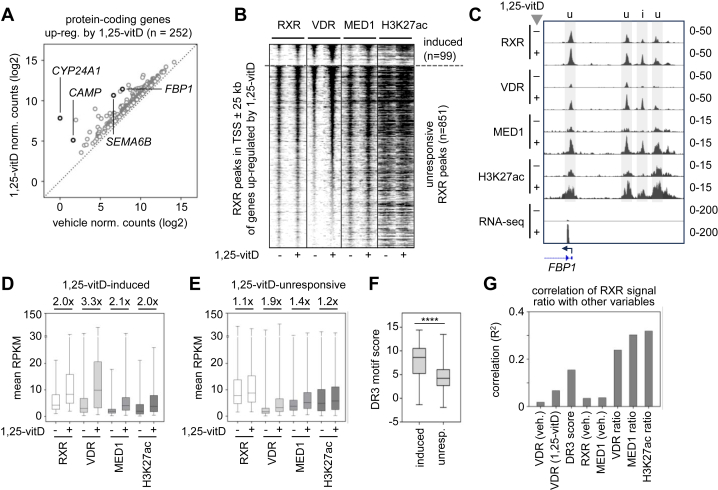
**Binding profile of RXR, VDR, MED1, and H3K27ac at TSS ± 25 kb of genes upregulated by 1,25-vitD**. *A*, scatter plot showing normalized mRNA levels in PMA-THP-1 cells treated with 1,25-vitD for 6 h relative to vehicle. Representative target genes are labeled on the plot. *B*, RD plot displaying signals within a ±2 kb window centered on RXR peak summits. Cells were treated with vehicle or 1,25-vitD for 1 h (RXR and VDR), 2 h (MED1), or 6 h (H3K27ac). RXR peaks within TSS ± 25 of genes upregulated by 1,25-vitD were classified as 1,25-vitD-induced (FC > 1.5, n = 99) or 1,25-vitD-unresponsive (n = 851). Two 1,25-vitD–reduced RXR peaks (FC < 0.66) were identified but are not shown in the RD plot. *C*, IGV snapshot of a representative gene, with tracks displaying normalized ChIP-seq and RNA-seq data. The 1,25-vitD-unresponsive and 1,25-vitD-induced RXR peaks are indicated with “u” and “i,” respectively. *D*–*E*, box-and-whisker plots showing ChIP-seq signal intensities for the indicated RXR peak sets identified in TSS ± 25 kb of upregulated genes. The same scales were used for 1,25-vitD–induced and 1,25-vitD–unresponsive RXR peaks for better comparison. Numbers above the box plots indicate the ratio of median ChIP-seq signal in 1,25-vitD–treated *versus* vehicle-treated cells. *F*, box-and-whisker plots of DR3 motif scores in 1,25-vitD–induced and 1,25-vitD–unresponsive RXR peak sets; ∗∗∗∗, *p* < 0.0001 (using an unpaired two-tailed *t* test). unresp., unresponsive. *G*, bar graph showing correlations between RXR peak ligand responsiveness and other features. For each peak, the RXR signal ratio (1,25-vitD *versus* vehicle) and corresponding feature values (*e*.*g*., RPKM in vehicle, signal ratios) were calculated, and correlation analysis was performed across all peaks. H3K27ac, Histone H3 acetylated at lysine 27; IGV, integrative genomics viewer; TSS, transcription start site.

Significantly upregulated genes (FDR < 0.05 and FC > 1.5) by 1,25-vitD after 6 h were identified by RNA-seq. The resulting gene list contained 252 protein-coding genes, including well-characterized VDR targets (Fig. 4*A* and Table S4). Previous studies have shown that functionally validated enhancer-promoter interactions are highly enriched at short genomic distances, with interaction frequency gradually decreasing as distance increases, up to 100 kb (117, 118). In our study, RXR-binding regions within ± 25 kb of the transcription start site (TSS) of the upregulated genes were mapped to capture regulatory elements potentially involved in transcriptional regulation. The mapped RXR-binding regions (n = 952) were classified based on 1,25-vitD–induced changes in RXR occupancy. This analysis identified 1,25-vitD–unresponsive (n = 851), 1,25-vitD–induced (n = 99), and 1,25-vitD–reduced (n = 2) RXR peaks in proximity to the upregulated genes (Fig. 4, *B* and *C* and Table S5).

The mapped 1,25-vitD–unresponsive and 1,25-vitD–induced RXR-binding regions were compared for VDR and MED1 binding, H3K27ac signals, and DR3 motif enrichment. (The two 1,25-vitD–reduced RXR peaks were not involved in this analysis.) We found that 1,25-vitD–induced RXR peaks exhibited higher fold induction of VDR, MED1, and H3K27ac signals; and basal VDR levels were also higher (Fig. 4, *D* and *E*). Notably, many 1,25-vitD–unresponsive RXR regions exhibited increased VDR occupancy upon ligand treatment, and the MED1 and H3K27ac data indicated that they were often associated with ligand-dependent enhancer activation (Fig. 4*E*). Notably, our results demonstrated that increases in VDR occupancy were higher than increases in RXR occupancy upon 1,25-vitD treatment in both sets (Fig. 4, *E*, *B*–*D*). 1,25-vitD–induced RXR peaks displayed stronger DR3 motif scores, reflecting a higher match to the canonical VDR-RXR binding motif (Fig. 4*F*). We also evaluated the correlation between 1,25-vitD-responsiveness of the mapped RXR peaks (n = 952; RXR occupancy signal ratio in 1,25-vitD *versus* vehicle) and other features. RXR signal ratios weakly correlated with DR3 motif score, and VDR, MED1, and H3K27ac signal ratios; no correlation was observed with other features, such as basal VDR or RXR levels (Fig. 4*G*). The results presented in Figure 4, *B*–*E* collectively suggested that both 1,25-vitD–induced and 1,25-vitD–unresponsive RXR peaks participated in transcriptional regulation.

### 1,25-vitD has different effects on VDR *versus* RXR occupancy at the same genomic regions

**Figure 5:**
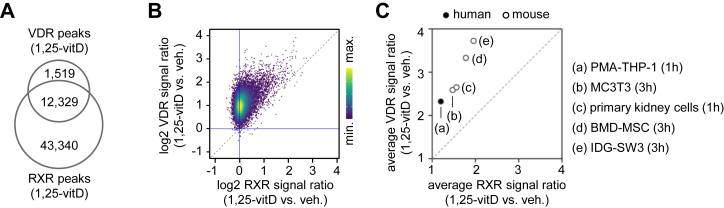
**Changes in RXR and VDR occupancy in response to 1,25-vitD treatment**. *A*, overlap of RXR and VDR ChIP-seq peaks in PMA-THP-1 cells treated for 1 h with 1,25-vitD. *B*, ligand-induced changes in RXR and VDR binding shown in a pseudocolor scatter plot. At common RXR and VDR binding regions (n = 12,329), occupancy signals were determined, signal ratios (1,25-vitD versus vehicle) were calculated for RXR and VDR, and the values were plotted against each other. *C*, ligand-induced changes in RXR and VDR binding across five studies. At common RXR and VDR binding regions, occupancy signals were determined, average signal ratio (1,25-vitD versus vehicle) were calculated for RXR and VDR, and the values were plotted against each other. BMD-MSC, bone marrow-derived mesenchymal stem cells; IDG-SW3, osteocytogenic cell line; MC3T3, osteoblast precursor cell line.

We observed that 1,25-vitD–induced increases in VDR binding occurred even at unresponsive RXR-binding regions (Fig. 4*B*), raising the question of whether the differences in RXR and VDR binding dynamics represent a general phenomenon. To compare ligand-induced increases in RXR and VDR occupancy not only in the TSS ± 25 kb of genes regulated by 1,25-vitD, but also globally, the analysis was extended to all common binding regions of RXR and VDR. For this comparison, all RXR peaks overlapping with VDR peaks in PMA-THP-1 cells were identified (n = 12,329, Fig. 5*A*) and the signal ratio (1,25-vitD *versus* vehicle) was calculated and compared for RXR and VDR at these regions. The results revealed a systematic shift toward VDR, suggesting that the observed difference in the ligand-induced changes in RXR and VDR occupancy represents a global genomic phenomenon (Fig. 5*B*).

To assess whether this shift also occurs in independent datasets from other studies, we screened published studies containing both RXR and VDR ChIP-seq data under vehicle and 1,25-vitD treatment conditions. Applying the same criteria used for selecting studies for meta-analysis in Figure 1, four studies in murine cell types were included (40, 65, 66, 67). The raw datasets were downloaded and re-analyzed, and the signal ratios (1,25-vitD *versus* vehicle) for RXR and VDR at co-occupied regions were calculated and compared for each dataset. Notably, the signal ratios across the four cell types varied for both VDR and RXR. These differences may be due to species-specific factors (mouse versus human), receptor expression levels, endogenous ligands, or technical variation. Despite these differences, a consistent shift toward VDR was detected in all four datasets, similar to our finding in PMA-THP-1 cells. The average of VDR signal ratios was higher than the average of RXR signal ratios (Fig. 5*C* and Fig. S5, *A*–*B*). Taken together, these analyses demonstrated that 1,25-vitD exerted distinct effects on VDR and RXR occupancy, indicating that ligand-driven changes in binding dynamics could differ between dimerization partners even at the same genomic loci.

### Treatments with “cocktails” containing multiple agonists cause only minor global changes in RXR occupancy

**Figure 6:**
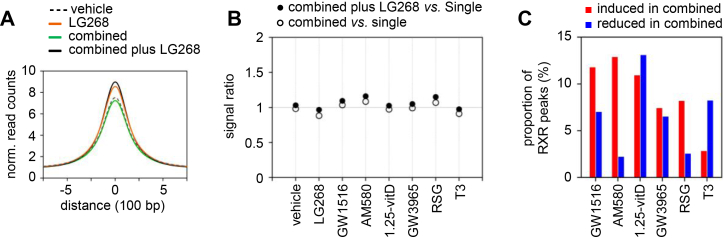
**The effects of ligand co-administration on RXR occupancy**. *A*, normalized read counts within ± 750 bp of RXR peak summits in vehicle- and ligand-stimulated PMA-THP-1 cells. All six agonists of RXR partners (combined) or the six agonists together with LG268 (combined plus LG268) were compared with vehicle and LG268 alone. *B*, signal ratios for combined and combined plus LG268 treatments relative to the corresponding single-ligand treatments are shown. Signals were calculated at RXR binding regions identified under each treatment condition, and the ratio of the signal in the combined or combined plus LG268 condition to that in the single-ligand treatment condition was determined. *C*, the proportions of RXR peaks that were induced (RXR signal ratio > 1.5) or reduced (RXR signal ratio < 0.66) in cells subjected to combined treatment relative to the indicated single treatments.

We examined RXR occupancy after co-administration of multiple ligands for 2 h using ChIP-seq and compared the results with the RXR occupancy following single-ligand treatments. We tested “cocktails” containing six agonists of RXR partner receptors (hereafter, combined) or the same six agonists together with LG268 (combined plus LG268). In the initial analysis, we calculated a consensus RXR peak set for vehicle, LG268, combined, and combined plus LG268 (peaks detected in at least two samples) and compared RXR occupancy across these conditions (Fig. 6*A*). LG268 alone induced a stronger increase in global RXR binding than the six partner-receptor agonists combined. Next, we performed an analysis analogous to that in Figure 2*E*, calculating RXR signal ratios for the combined and combined plus LG268 treatments relative to the corresponding single-ligand treatments for each reproducible RXR peak set. Only moderate increases in overall RXR binding were detected compared with the corresponding single-ligand stimulations (Fig. 6*B*).

The redistribution of RXR binding was determined by the identification of RXR peaks with increased or decreased occupancy (RXR signal ratio in combined or combined plus LG268 *versus* single ligands) using cutoffs of >1.5 for increases and <0.66 for decreases. We first performed this analysis on the reproducible RXR peak sets. Across all conditions, the proportion of RXR peaks exhibiting increased or decreased binding upon combined treatment did not exceed 15% (Fig. 6*C*). The largest induction and reduction occurred when RXR occupancies after the combined or combined plus LG268 treatment were compared with occupancies after AM580 and 1,25-vitD treatment, respectively (Fig. 6*C* and Fig. S6*A*). A similar analysis of the ligand-responsive exploratory RXR peak sets (peak sets indicated in Fig. S3*A*) showed that the majority of ligand-induced RXR peak sets remained unchanged following combined ligand treatment, whereas ligand-reduced peaks were more frequently affected by combined ligand treatment (Fig. S6, *B*–*C*). Collectively, these results indicated that co-activation of multiple RXR partners induced only minor global changes in RXR chromatin occupancy, and redistribution occurred at a limited subset of RXR-binding regions in PMA-THP-1 cells.

### Combined ligand treatment has a limited impact on RXR occupancy and transcriptional response compared to 1,25-vitD

We aimed to examine the association between changes in RXR binding and changes in gene expression induced by co-activation of multiple RXR partners. For this analysis, we focused on 1,25-vitD treatment as a reference to assess RXR binding and gene regulation in response to combined ligand treatment.

**Figure 7:**
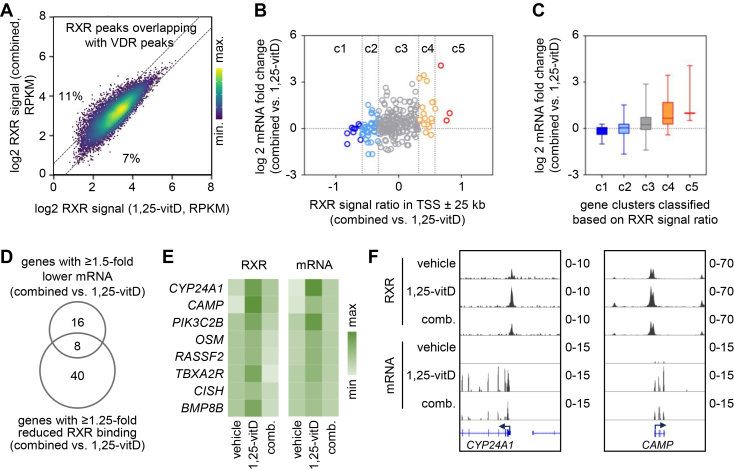
**The effects of combined versus 1,25-vitD treatment on RXR occupancy and mRNA level**. *A*, scatter plot showing RXR signals in PMA-THP-1 cells treated with the combined treatment with agonists for the six RXR partners (combined) compared with 1,25-vitD. RXR peaks overlapping with VDR peaks are displayed. Dotted lines indicate RXR ratios (combined *versus* 1,25-vitD) of 1.5 and 0.66. *B*, scatter plot showing the FCs (combined *versus* 1,25-vitD) in mRNA levels and the RXR signal ratio (combined versus 1,25-vitD) within the TSS ± 25 kb of the corresponding genes. Genes upregulated by 1,25-vitD and associated with at least one RXR peak in their TSS ± 25 kb are shown (n = 241). Genes were clustered and color-coded based on the RXR ratio (R) as follows: c1, R ≤ 0.66; c2, 0.66 < R ≤ 0.8; c3, 0.8 < R ≤ 1.25; c4, 1.25 < R ≤ 1.5; c5, R > 1.5. *C*, box-and-whisker plots showing the mRNA FCs (combined versus 1,25-vitD) for the five gene clusters (c1-c5). *D*, comparison between the two subsets of genes upregulated by 1,25-vitD. The first subset includes genes whose mRNA levels were at least 1.5-fold lower in cells treated with the combined treatment compared with cells treated with 1,25-vitD. The second subset includes genes associated with at least a 1.25-fold reduction in RXR binding (combined versus 1,25-vitD) within the TSS ± 25 kb region. *E*, heatmaps of RXR occupancy in TSS ± 25 kb and mRNA levels of genes common to both subsets indicated in (D) (n = 8 genes). *F*, Integrative Genomics Viewer snapshots of two representative genes upregulated by 1,25-vitD, in which the combined treatment relative to 1,25-vitD led to lower mRNA levels and reduced RXR binding. Tracks show normalized RXR ChIP-seq and RNA-seq data within the TSS ± 10 kb regions. TSS, transcription start site.

We compared RXR occupancy between cells treated with 1,25-vitD alone and cells treated with the combination of six agonists using the RXR peak set overlapping with VDR peaks. We found that RXR signals were reduced or induced in only a small proportion of RXR peaks (7% and 11%, respectively) upon combined treatment compared with 1,25-vitD treatment (Fig. 7*A*). These percentages were 12% and 25% when the combined plus LG268 *versus* 1,25-vitD treatment conditions were compared (Fig. S7*A*). Notably, other RXR heterodimers or RXR homodimers may also occupy the regions where RXR overlaps with VDR. This assumption was confirmed by our motif analysis (Fig. S7*B*). We calculated the prevalence of DR0-DR5 motifs in this set; many of the RXR peaks overlapping VDR peaks contained DR3 motifs and motifs preferred by other RXR partners, often in combination with the DR3 motif. Therefore, the changes in RXR signals in these regions may reflect the net effects of ligand treatment on various RXR dimers, as well as the potential redistribution of RXR between genomic regions. We performed a similar analysis on the RXR peak set overlapping with RARα peaks determined in our previous study (64). This analysis revealed that combined treatment induced RXR occupancy in 18% of the peaks, while no peaks showed reduced occupancy compared with AM580 alone (Fig. S7*C*). When combined plus LG268 treatment was compared with AM580 alone, the percentage of induced RXR peaks was even higher (40% induced versus 1% reduced), suggesting that activation of permissive heterodimers and/or RXR homodimers may enhance RXR binding (Fig. S7*D*).

We investigated the relationship between RXR binding and transcriptional output. For this analysis, we integrated the ChIP-seq and RNA-seq data for genes upregulated by 1,25-vitD that contained at least one RXR peak within their TSS ± 25 kb (n = 241). Few 1,25-vitD–responsive genes (n = 11) were not associated with any RXR peak in these regions and were therefore not included in this analysis. Genes upregulated by 1,25-vitD were grouped into five clusters according to the RXR signal ratio (combined vs. 1,25-vitD only). This ratio was calculated by summing the RXR occupancy in peaks within the TSS ± 25 kb of the genes upon combined or 1,25-vitD treatments (Fig. 7, *B* and *C* and Table S6). A comparison of mRNA changes between these clusters revealed no overall decrease in expression for genes with reduced RXR binding in response to combined treatment relative to treatment with 1,25-vitD only. In contrast, genes associated with increased RXR occupancy exhibited moderately elevated mRNA levels in response to combined treatment relative to treatment with 1,25-vitD only (Fig. 7*C*).

We compared the overlap between genes showing reduced mRNA levels (≥1.5-fold decrease) and genes exhibiting reduced RXR binding in TSS ± 25 kb (≥1.25-fold reduction) in response to combined treatment. Only eight genes were common to both subsets (Fig. 7, *D*–*F*). These results indicate that any interference caused by the combined ligand treatment is modest at the transcriptional level (∼9.9%, 24 genes of the 241 protein-coding genes upregulated by 1,25-vitD), with only a very small subset (∼3.3%, eight genes) exhibiting concurrent reductions in both RXR binding and mRNA expression.

## Discussion

### Characterization of ligand-induced RXR chromatin binding using ChIP-seq

Multiple ChIP-seq studies have elucidated the RXR binding landscape in various cell types following stimulation with agonists of RXR or its heterodimeric partners (26, 38, 39, 40, 65, 66, 67, 68, 69, 119, 120, 121, 122, 123, 124, 125, 126, 127, 128). Although all available studies were systematically evaluated, only one RXR ChIP-seq dataset (38) qualified for inclusion in the meta-analysis, whereas the others were excluded due to the absence of control samples, treatment durations exceeding 24 h, or other predefined criteria. In our study, RXR binding was examined under four experimental conditions, and other treatments (*e*.*g*., RXR inverse agonist, 9-cis-RA, or diverse ligand combinations) were not used in this work. These four conditions included: (i) no exogenous ligands (vehicle-treated), (ii) the RXR agonist LG268, (iii) single RXR partner agonists, and (iv) “cocktails” containing multiple agonists. The RXR binding results under these four conditions are discussed below.

### RXR DNA occupancy in the absence of exogenous agonists

We detected 48,260 reproducible RXR peaks in the vehicle condition (Fig. 2*C*), the highest number of RXR peaks reported to date in any cell type. Motif analysis (Fig. 3*C*) revealed enrichment of DR0-DR5-type motifs in the top RXR peaks in vehicle condition, indicating that various RXR heterodimers bind to DNA. Although no exogenous agonists were added for the vehicle condition, low levels of endogenous ligands may have been present. Previous studies demonstrated that RXR recognizes specific DNA sequences *via* its DNA-binding domain *in vitro* without added ligand (8, 129, 130, 131, 132). This indicates that DNA binding is an intrinsic property of the receptor. *In vivo*, TFs do not bind all putative DNA motifs because the local chromatin environment and interacting proteins (dimerization partners, cofactors, other TFs) largely influence the binding, explaining why RXR occupies a small proportion of canonical DR0-DR5 motifs.

### Effects of RXR-specific agonist LG268 on RXR DNA binding

In PMA-THP-1 cells, LG268 produced the most pronounced effect on RXR chromatin binding among the treatments tested (Fig. 2, *C* and *E*). This observation aligns with previous findings in mouse bone marrow–derived macrophages, where LG268 induced greater changes in RXR occupancy than rosiglitazone (RSG) or GW3965 (38). Similarly, live-cell fluorescence correlation spectroscopy measurements in HeLa cells expressing EGFP-RXR demonstrated that only RXR ligands (LG268 or 9-cis-RA), not partner NR ligands (AM580 or RSG), increased the fraction of RXR molecules in the chromatin-bound state (26). Changes in RXR occupancy induced by RXR agonists at genomic loci may be explained by at least four nonmutually exclusive mechanisms. The four mechanisms include the following: (i) increased intrinsic DNA-binding affinity *via* ligand-induced conformational changes; (ii) ligand-induced tetramer dissociation that increases the pool of DNA-binding–competent RXR dimers(131, 133); (iii) enhanced RXR dimerization and prolonged chromatin retention; and (iv) cofactor recruitment that stabilizes RXR-DNA binding. The motif analysis of the LG268-induced RXR peak set revealed that DR1-type direct repeats were approximately two-fold more enriched compared to other motif types. This DR1 bias was less pronounced when top RXR peaks in vehicle condition were analyzed (Fig. 3*C*). These results indicated that LG268 treatment caused a shift in the RXR-binding profile, reflecting an increased preference for DR1-containing regions. The shift is likely driven by the enhanced formation of RXR homodimers and/or heterodimers, which prefer DR1. This uneven redistribution of RXR across binding regions suggests that mechanisms iii and iv are important contributors to the LG268-induced changes in RXR occupancy.

### Effects of partner agonists on RXR binding

Changes in RXR chromatin binding upon partner-agonist treatment must be interpreted as indirect effects, mediated *via* the ligand’s action on the partner receptor and, in turn, on RXR. To interpret the effects of partner agonists on RXR binding, two key issues must be considered. First, the effect of the ligand on the DNA binding of its own receptor. Second, the relationship of ligand-induced changes in the binding of the partner receptor and RXR.

Regarding the first issue, our meta-analysis indicated that ligand stimulation typically only slightly alters overall occupancy, with the exception that VDR produces the largest overall binding increases (Figs. 1*B*). Notably, only limited studies were available for other RXR partners. Among RAR ChIP-seq studies meeting our selection criteria (n = 4), only our previous work reported a modest global increase in RARα occupancy (ratio 1.15; Fig. 1*B*, Table S1) (64). Pronounced RAR cistrome remodeling previously observed in stem-cell models may reflect a combination of direct receptor-mediated effects and broader changes associated with retinoic acid–induced differentiation (39, 119). The ligand-induced DNA-binding results are often controversial. For example, RAR shows both ligand-induced (134) and ligand-unresponsive binding (135, 136, 137) in studies using EMSA. Live-cell FRET and fluorescence (cross-) correlation spectroscopy demonstrated ligand-induced DNA binding and RAR-RXR dimerization (25, 28, 29). However, single-molecule tracking did not detect a change in RAR-RXR DNA binding upon ligand stimulation (32), and ChIP-seq also showed only a modest global increase in RARα occupancy (64). The potential mechanisms for increased DNA binding include the following: increased intrinsic DNA-binding affinity, ligand-induced dimerization, and/or cofactor recruitment that stabilizes DNA binding (23, 26, 27, 29, 138, 139). An additional potential mechanism described for VDR, namely ligand-induced nuclear translocation, analogous to that of GR, likely contributes to increased DNA binding (27, 29, 140). It is not clear whether this explains why ligand stimulation produces the largest overall binding increases of VDR, as shown by ChIP-seq data from previously published studies and our own datasets (Figs. 1*B* and 4*B*).

Regarding the second issue, the effect of any partner ligand on RXR is not expected to surpass its effect on the ligand’s own receptor. The relationship of ligand-induced changes in DNA binding of RXR and its dimerization partner is largely unexplored. Even in studies where ChIP-seq data for both RXR and its partner are available following treatment with the same ligand, such direct comparisons have not been performed. We compared the effect of 1,25-vitD on the DNA binding of RXR and VDR. Our results showed that treatment with 1,25-vitD markedly enhanced VDR DNA-binding, but the corresponding increase in RXR occupancy was substantially smaller (Fig. 5*B*). Similar patterns were observed across multiple independent studies in human and mouse cells (Fig. 5*C* and Fig. S5). This differential response demonstrates that ligand-induced VDR binding was not coupled to proportional increases in RXR occupancy. Our data suggested that the residence times of VDR and RXR on DNA are not necessarily coupled, meaning that ligand-driven changes in the binding dynamics of one partner can differ from those of another. However, the RXR occupancy was still influenced by 1,25-vitD (Fig. 3*B*). Increased RXR occupancy upon partner ligand is likely attributed to the previously mentioned mechanisms (increased heterodimerization and coactivator recruitment).

### Effects of ‘cocktails’ containing multiple agonists on RXR binding

In our study, simultaneous activation of multiple RXR partner pathways caused only modest changes in RXR occupancy (Fig. 6, *B* and *C*), indicating that the RXR pool is sufficient to support multiple partners in macrophage-like cells. Macrophages are immune cells that detect and integrate various environmental cues, being well equipped with receptors that recognize pathogen- and damage-associated molecular patterns, cytokines, and NR ligands (5). In THP-1 cells, several NRs are expressed, but *RXR* mRNA levels exceed those of its partners, similar to primary macrophages (Fig. 2, *A* and *B*). This suggests that RXR may be present in stoichiometric excess relative to its partners in these cell types, similar to what has been previously observed in the U2OS cell line (32). Whether RXR is limiting in other cell types with lower RXR levels or higher partner expression remains to be determined. We found that only ∼3.3% of the protein-coding genes upregulated by 1,25-vitD exhibited both decreased RXR binding and reduced expression in response to combined treatment relative to 1,25-vitD treatment alone (Fig. 7*D*). These genes generally showed low FCs. Notable exceptions were *CAMP* and *CYP24A1*, two well-known 1,25-vitD target genes, which were expressed at very low levels in vehicle-treated cells (Fig. 4*A*). Our findings indicate that interference with 1,25-vitD–induced gene regulation during simultaneous activation of multiple NR pathways is not necessarily due to RXR sequestration. Instead, other NR cross-talk mechanisms, such as competition for DNA response elements, cofactor titration, or indirect signaling repression, likely contribute to this interference (52). We cannot exclude the possibility that the transcriptional responses are affected or compensated for by alternative gene regulatory mechanisms.

From a design perspective, sharing any module among pathways can make the system prone to interference, as module availability may become limiting when multiple pathways are simultaneously activated. Notably, this concern is not limited to RXR but also applies to other components involved in transcription, including cofactors and RNA polymerase II, which are shared across pathways. Our results suggest that RXR functions as a nonlimiting, shared component across multiple pathways in macrophage-like cells. In our experimental setup, many RXR-dependent pathways are fully activated; even under this “overloaded” condition, pathway interference detected at the mRNA level is minimally attributable to RXR availability.

## Experimental procedures

### Cell culture and ligand treatment

The human monocytic cell line, THP-1, was obtained from the American Type Culture Collection. Cells were cultured in a humidified atmosphere at 37 °C and 5% CO_2_ in RPMI-1640 medium (Gibco) supplemented with 10% fetal bovine serum (Biosera), 0.05 mM 2-mercaptoethanol (Gibco), 1% penicillin-streptomycin solution (Sigma-Aldrich), and 1 mM sodium-pyruvate (Gibco). Cells were passaged every 3 days, and the cell density after each passage was approximately 250,000 cells/ml. Cells were stimulated with 20 nM PMA (Sigma-Aldrich) for 16 h to differentiate THP-1 cells into macrophage-like cells (PMA-THP-1 cells). After differentiation, PMA-THP-1 cells were maintained in phenol red–free RPMI medium (Gibco) supplemented with 10% charcoal-stripped fetal bovine serum of South American origin (Biowest) and 1% penicillin-streptomycin solution for 30 min. Cells were then stimulated with 1:1 DMSO-ethanol (vehicle) or agonists for RARα (100 nM AM580, BioVision), VDR (100 nM calcitriol or 1α,25-dihydroxyvitamin D3 (1,25-vitD), Sigma-Aldrich), PPARδ (100 nM GW501516 (GW1516), Sigma-Aldrich), PPARγ (1 μM RSG, Cayman chemical), LXR (1 μM GW3965, Sigma-Aldrich), TR (100 nM 3,3′,5-Triiodo-L-thyronine (T_3_), Sigma-Aldrich), RXR (1 μM LG100268 (LG268), Sigma-Aldrich), or a combination of all of these agonists with or without LG268 (referred to as “combined” and “combined plus LG268,” respectively). Cells were harvested at different times depending on the experiment.

### RNA-seq experiments and data analysis

For RNA-seq experiments, PMA-THP-1 cells were stimulated for 6 h with vehicle or ligands, and total RNA was isolated using a Quick-RNA Miniprep Kit (ZYMO research). The quality of RNA was verified on an Agilent BioAnalyzer using an Eukaryotic Total RNA Nano Kit. RNA-seq libraries were prepared from total RNA using an Ultra II RNA Sample Prep kit (New England BioLabs). Sequencing was performed on the Illumina NextSeq 2000 platform using single-end 75-cycle sequencing. Nine RNA-seq datasets were generated, including three replicates for vehicle, 1,25vitD, or combined agonist-treated cells. In addition, the RNA-seq data generated in our previous work were partly used in this study (64), which is available at GSE246309 in the NCBI Gene Expression Omnibus (GEO) http://www.ncbi.nlm.nih.gov/geo/. Reads were aligned to hg38 (HISAT2). BAM files were imported into the Strand NGS program (https://www.strand-ngs.com/) and processed using the software’s integrated DESeq algorithm for quantification and normalization. Genes expressed at low levels in all conditions were excluded. Differentially expressed genes were identified using ANOVA with Benjamini–Hochberg correction and a Tukey *post hoc* test (*p* < 0.05). A 1.5 cutoff was used to determine the upregulated genes. Protein-coding genes were extracted using the HUGO Gene Nomenclature Committee (HGNC) protein-coding gene list (https://www.genenames.org/download/statistics-and-files/). A Gene Transfer Format file (GRCh38.p14) was used to determine the TSS of the genes.

### ChIP-seq experiments

The ChIP-seq experiments were performed as previously described (141, 142, 143). Briefly, 3 or 10 million PMA-THP-1 cells were treated with vehicle or ligands for 1, 2, or 6 h for RXR and Histone H3 acetylated at lysine 27 (H3K27ac) ChIP-seq experiments. The cells were cross-linked with 2 mM disuccinimidyl glutarate (Sigma-Aldrich) for 40 min and 1% methanol-free formaldehyde (Thermo Fisher Scientific) for 10 min. Cross-linking was terminated by treating with 0.125 M glycine (Sigma-Aldrich) for 10 min. The ChIP lysis buffer (150 mM NaCl, 1 mM EDTA, pH 8, 20 mM Tris–HCl (pH 8), 1% Triton X-100, and 0.1% SDS) was supplemented with protease inhibitors (cOmplete Mini EDTA-free protease inhibitor cocktail, Roche). The chromatin was sheared by sonication (Diagenode Bioruptor Plus) and immunoprecipitated overnight using antibodies against RXRα (21218-1-AP, Proteintech) and H3K27ac (ab4729, Abcam). Chromatin–antibody complexes were pulled down with magnetic beads (Protein A or G Dynabeads, Thermo Fisher Scientific), washed, and eluted. Eluted complexes were decrosslinked overnight and purified using NucleoSpin Gel and a PCR Clean-up Kit (Macherey-Nagel). ChIP-DNA was quantified using a Qubit fluorimeter. Indexed complementary DNA libraries were prepared from 1 to 10 ng of ChIP-DNA using an Ovation Ultralow System V2 (Tecan), according to the manufacturer's instructions. Libraries were sequenced on the Illumina NextSeq 2000 platform using single-end 75-cycle sequencing. The following 28 ChIP-seq datasets were generated: two replicates of RXR ChIP-seq from PMA-THP-1 cells treated with vehicle, AM580, 1,25-vitD, GW1516, RSG, GW3965, T_3_, LG268, combined ligands, or combined plus LG268 for 2 h (n = 20); two replicates of RXR ChIP-seq from PMA-THP-1 cells treated with vehicle and 1,25-vitD for 1 h (n = 4); and two replicates of H3K27ac ChIP-seq from PMA-THP-1 cells treated with vehicle or 1,25-vitD for 6 h (n = 4). The MED1, RARα, and VDR ChIP-seq datasets generated as part of our previous work were used in this study (64), available at GSE246308 in NCBI - GEO http://www.ncbi.nlm.nih.gov/geo/.

### ChIP-seq data analysis

The ChIP-seq data were analyzed using our ChIP-seq analysis pipeline (144), as described previously (143). Model-based analysis of ChIP-seq version 2 (145) was used for peak calling with the following specific parameters: q value cutoff = 0.001 and subpeaks deconvolved within each peak (call-summits). Artifacts were removed using the ENCODE blacklist (146). Regions with accession prefixes of NW and NT were excluded; only regions starting with NC (complete genomic assembly) were used for the analyses. The overlapping peak sets between the two replicates were identified using intersectBed and merged with mergeBed (bedtools) and subsequently retained for further analysis. Normalized tag counts (expressed as reads per kilobase per million mapped reads (RPKM)) were calculated using bamtools, bedtools, and awk. Integrative Genomics Viewer (Broad Institute) (147) was used for data browsing and creating representative snapshots. The values in the genome coverage files (BedGraphs) were converted into tile data format files using igvtools with the “toTDF” option. Read distribution plots were generated by annotatePeaks.pl (HOMER) (148), using tag directories and bed files. The histograms were visualized with Java TreeView. Statistical analyses and plotting of graphs were performed using GraphPad Prism.

### Analysis of ligand-induced changes in ChIP-seq datasets and overlap between genomic regions

The R Bioconductor package, DiffBind (version 3.16), with the edgeR tool, was used to identify RXR peaks with changes in the RXR occupancy (149). Peaks with a FC greater than 1.5 or less than 0.66 were classified as ligand-induced or ligand-reduced exploratory peak sets, respectively, and the remaining peaks were classified as ligand-unresponsive peaks. Volcano plot showing the RXR signals in LG268 *versus* vehicle-treated samples (FC and FDR) was generated using DiffBind. Heatmaps were generated using either frequency (percentage) values or median-normalized values, as indicated for each heatmap. The intersectBed (bedtools) was used to determine the overlap between RXR ChIP-seq peaks in cells treated with vehicle and ligand(s) and between the RXR and VDR or RARα ChIP-seq peaks.

### Meta-analysis of ChIP-seq datasets

In step 1, ChIP-seq datasets in the Sequence Read Archive (SRA) were retrieved using combinations of keywords, including the name of the NR of interest, "ChIP," and the species "human" and "mouse." Using the "Send to File" and "RunInfo download" options, a .csv file was generated for each NR. In step 2, ChIP-seq samples were selected for analysis. For entries with a GEO accession (GSM code), additional metadata was downloaded from GEO. Samples unrelated to ChIP-seq (such as RNA-seq datasets) were excluded, and entries that included the name of the NR in the "antibody" or "chip_antibody" metadata fields were retained. BioProjects for analysis were selected based on the following three criteria: (i) both control and ligand-stimulated samples were included, with treatment duration no longer than 24 h, (ii) must be associated with a published article, and (iii) only one time point (closest to 2 h), one type of ligand (the most commonly used), and one cell type per study were included in the analysis. In step 3, FASTQ files were downloaded. In step 4, primary data analysis was performed. The SRA run files belonging to the same SRA experiments (SRX) were merged, and the sequences were aligned using our in-house pipeline to generate BAM files. In step 5, peak calling was performed, as described earlier, and consensus peak sets were calculated. Consensus regions for each NR were determined as follows: within each study, the filtered narrowPeak files for individual samples were merged using MergeBed. If only one replicate per treatment condition (control and ligand-stimulated samples) was available, the merged peaks were considered the consensus regions. If two or more replicates were available for each treatment condition, only those peaks present in at least two different samples were retained. Studies with fewer than 2000 consensus peaks were excluded. If more than 10 studies were qualified for a given NR, the most recent studies with at least two replicates per treatment condition were selected. If fewer than 10 such studies were available for a given NR, the most recent studies with one replicate per sample were included to reach a maximum of 10 studies for that NR. In step 6, RPKM values were calculated separately for each region in each study, and the results were compiled into a table. The average RPKM values between replicates (if any) of each treatment condition were calculated for each consensus region. The median RPKM values in control and ligand-stimulated samples and the ratio (ligand-stimulated veraus control) were calculated. DiffBind was used for correlation analysis of peak sets, and the resulting correlation heatmap was generated using the “pheatmap” package in R (version 1.0.13). Histograms showing normalized read counts were generated by annotatePeaks.pl (HOMER) (148) and visualized by GraphPad Prism.

### DNA motif analysis

The term “DNA motif” refers to a set of aligned sequences summarized by position weight matrices (PWMs) and visualized using motif logos. The occurrence of PWMs was evaluated in the peak sets in four steps. In step 1, the PWMs, including RAR-DR0 (GSE56893/Homer, motif 61), PPARa-DR1 (GSE47954/Homer, motif 290), RARg-DR2 (MA0859.2/Jaspar), VDR-DR3 (GSE22484/Homer, motif 394), LXR-DR4 (MA0494.1/Jaspar), and RARα-DR5 (GSE56893/Homer, motif 62), were obtained from the HOMER and Jaspar databases (http://homer.ucsd.edu/homer/motif/HomerMotifDB/homerResults.html and https://jaspar2018.genereg.net/collection/core/). In step 2, the PWM alignment scores were calculated using control sets containing randomly selected, size-matched genomic sequences from regions starting with NC (complete genomic assembly) (n = 5000). Alignment scores, reflecting sequence similarity with the PWM, were determined using annotatePeaks.pl (HOMER). The score thresholds (cutoffs) were defined as the scores giving 5% positive matches in the control set. In step 3, DNA motifs in the entire human genome were mapped using the PWMs and the computed score thresholds in scanMotifGenomeWide.pl (HOMER). In step 4, the prevalences of motifs in various RXR peak sets were determined using intersectBed (bedtools).

## Data availability

The data underlying this article are available in NCBI Gene Expression Omnibus (GEO) at http://www.ncbi.nlm.nih.gov/geo/ under the accession numbers GSE315571 (ChIP-seq) and GSE315572 (RNA-seq).

## Supporting information

This article contains supporting information.

## Conflict of interest

The authors declare that they have no conflicts of interest with the contents of this article.

## Supporting information

Supplementary Tables legends

Supplementary Table S1 meta analysed datasets

Supplementary Table S2 list of human nuclear receptors and features

Supplementary Table S3 exploratory RXR peak sets

Supplementary Table S4 differentially expressed genes by vitD

Supplementary Table S5 RXR in TSS+_25kb of vitD upreg genes

Supplementary Table S6 vitD target genes mRNA vs RXR

## References

[1] Mangelsdorf D.J., Borgmeyer U., Heyman R.A., Zhou J.Y., Ong E.S., Oro A.E. (1992). Characterization of three RXR genes that mediate the action of 9-cis retinoic acid. Genes Dev..

[2] Szanto A., Narkar V., Shen Q., Uray I.P., Davies P.J., Nagy L. (2004). Retinoid X receptors: X-ploring their (patho)physiological functions. Cell Death Differ..

[3] Mangelsdorf D.J., Evans R.M. (1995). The RXR heterodimers and orphan receptors. Cell.

[4] Germain P., Chambon P., Eichele G., Evans R.M., Lazar M.A., Leid M. (2006). International union of pharmacology. LXIII. Retinoid X receptors. Pharmacol. Rev..

[5] Nagy L., Szanto A., Szatmari I., Széles L. (2012). Nuclear hormone receptors enable macrophages and dendritic cells to sense their lipid environment and shape their immune response. Physiol. Rev..

[6] Dawson M.I., Xia Z. (2012). The retinoid X receptors and their ligands. Biochim. Biophys. Acta.

[7] Széles L., Póliska S., Nagy G., Szatmari I., Szanto A., Pap A. (2010). Research resource: transcriptome profiling of genes regulated by RXR and its permissive and nonpermissive partners in differentiating monocyte-derived dendritic cells. Mol. Endocrinol..

[8] Vivat-Hannah V., Bourguet W., Gottardis M., Gronemeyer H. (2003). Separation of retinoid X receptor homo- and heterodimerization functions. Mol. Cell Biol..

[9] Zhang X.K., Lehmann J., Hoffmann B., Dawson M.I., Cameron J., Graupner G. (1992). Homodimer formation of retinoid X receptor induced by 9-cis retinoic acid. Nature.

[10] Rühl R., Krzyżosiak A., Niewiadomska-Cimicka A., Rochel N., Szeles L., Vaz B. (2015). 9-cis-13,14-Dihydroretinoic acid is an endogenous retinoid acting as RXR ligand in mice. PLoS Genet..

[11] Heyman R.A., Mangelsdorf D.J., Dyck J.A., Stein R.B., Eichele G., Evans R.M. (1992). 9-cis retinoic acid is a high affinity ligand for the retinoid X receptor. Cell.

[12] Levin A.A., Sturzenbecker L.J., Kazmer S., Bosakowski T., Huselton C., Allenby G. (1992). 9-cis retinoic acid stereoisomer binds and activates the nuclear receptor RXR alpha. Nature.

[13] Lefebvre P., Benomar Y., Staels B. (2010). Retinoid X receptors: common heterodimerization partners with distinct functions. Trends Endocrinol. Metab..

[14] Evans R.M., Mangelsdorf D.J. (2014). Nuclear receptors, RXR, and the big bang. Cell.

[15] Alexander S.P.H., Cidlowski J.A., Kelly E., Mathie A.A., Peters J.A., Veale E.L. (2023). The concise guide to PHARMACOLOGY 2023/24: nuclear hormone receptors. Br. J. Pharmacol..

[16] Umesono K., Murakami K.K., Thompson C.C., Evans R.M. (1991). Direct repeats as selective response elements for the thyroid hormone, retinoic acid, and vitamin D3 receptors. Cell.

[17] Glass C.K., Holloway J.M., Devary O.V., Rosenfeld M.G. (1988). The thyroid hormone receptor binds with opposite transcriptional effects to a common sequence motif in thyroid hormone and estrogen response elements. Cell.

[18] Moutier E., Ye T., Choukrallah M.A., Urban S., Osz J., Chatagnon A. (2012). Retinoic acid receptors recognize the mouse genome through binding elements with diverse spacing and topology. J. Biol. Chem..

[19] Picard D., Yamamoto K.R. (1987). Two signals mediate hormone-dependent nuclear localization of the glucocorticoid receptor. EMBO J..

[20] Beato M., Klug J. (2000). Steroid hormone receptors: an update. Hum. Reprod. Update.

[21] Glass C.K., Rosenfeld M.G. (2000). The coregulator exchange in transcriptional functions of nuclear receptors. Genes Dev..

[22] McKenna N.J., O'Malley B.W. (2002). Combinatorial control of gene expression by nuclear receptors and coregulators. Cell.

[23] Nagy L., Schwabe J.W. (2004). Mechanism of the nuclear receptor molecular switch. Trends Biochem. Sci..

[24] McNally J.G., Müller W.G., Walker D., Wolford R., Hager G.L. (2000). The glucocorticoid receptor: rapid exchange with regulatory sites in living cells. Science.

[25] Brazda P., Szekeres T., Bravics B., Tóth K., Vámosi G., Nagy L. (2011). Live-cell fluorescence correlation spectroscopy dissects the role of coregulator exchange and chromatin binding in retinoic acid receptor mobility. J. Cell Sci..

[26] Brazda P., Krieger J., Daniel B., Jonas D., Szekeres T., Langowski J. (2014). Ligand binding shifts highly mobile retinoid X receptor to the chromatin-bound state in a coactivator-dependent manner, as revealed by single-cell imaging. Mol. Cell Biol..

[27] Fadel L., Rehó B., Volkó J., Bojcsuk D., Kolostyák Z., Nagy G. (2020). Agonist binding directs dynamic competition among nuclear receptors for heterodimerization with retinoid X receptor. J. Biol. Chem..

[28] Rehó B., Lau L., Mocsár G., Müller G., Fadel L., Brázda P. (2020). Simultaneous mapping of molecular proximity and comobility reveals agonist-enhanced dimerization and DNA binding of nuclear receptors. Anal. Chem..

[29] Rehó B., Fadel L., Brazda P., Benziane A., Hegedüs É., Sen P. (2023). Agonist-controlled competition of RAR and VDR nuclear receptors for heterodimerization with RXR is manifested in their DNA binding. J. Biol. Chem..

[30] Voss T.C., Schiltz R.L., Sung M.H., Yen P.M., Stamatoyannopoulos J.A., Biddie S.C. (2011). Dynamic exchange at regulatory elements during chromatin remodeling underlies assisted loading mechanism. Cell.

[31] Paakinaho V., Presman D.M., Ball D.A., Johnson T.A., Schiltz R.L., Levitt P. (2017). Single-molecule analysis of steroid receptor and cofactor action in living cells. Nat. Commun..

[32] Dahal L., Graham T.G., Dailey G.M., Heckert A., Tjian R., Darzacq X. (2024). Surprising features of nuclear receptor interaction networks revealed by live cell single molecule imaging. bioRxiv.

[33] Shlyueva D., Stampfel G., Stark A. (2014). Transcriptional enhancers: from properties to genome-wide predictions. Nat. Rev. Genet..

[34] Glass C.K., Natoli G. (2016). Molecular control of activation and priming in macrophages. Nat. Immunol..

[35] Daniel B., Nagy G., Horvath A., Czimmerer Z., Cuaranta-Monroy I., Poliska S. (2018). The IL-4/STAT6/PPARγ signaling axis is driving the expansion of the RXR heterodimer cistrome, providing complex ligand responsiveness in macrophages. Nucleic Acids Res..

[36] Czimmerer Z., Nagy Z.S., Nagy G., Horvath A., Silye-Cseh T., Kriston A. (2018). Extensive and functional overlap of the STAT6 and RXR cistromes in the active enhancer repertoire of human CD14+ monocyte derived differentiating macrophages. Mol. Cell. Endocrinol..

[37] Nielsen R., Pedersen T.A., Hagenbeek D., Moulos P., Siersbaek R., Megens E. (2008). Genome-wide profiling of PPARgamma:RXR and RNA polymerase II occupancy reveals temporal activation of distinct metabolic pathways and changes in RXR dimer composition during adipogenesis. Genes Dev..

[38] Daniel B., Nagy G., Hah N., Horvath A., Czimmerer Z., Poliska S. (2014). The active enhancer network operated by liganded RXR supports angiogenic activity in macrophages. Genes Dev..

[39] Chatagnon A., Veber P., Morin V., Bedo J., Triqueneaux G., Sémon M. (2015). RAR/RXR binding dynamics distinguish pluripotency from differentiation associated cis-regulatory elements. Nucleic Acids Res..

[40] Meyer M.B., Benkusky N.A., Sen B., Rubin J., Pike J.W. (2016). Epigenetic plasticity drives adipogenic and osteogenic differentiation of marrow-derived mesenchymal stem cells. J. Biol. Chem..

[41] Chanput W., Mes J.J., Wichers H.J. (2014). THP-1 cell line: an in vitro cell model for immune modulation approach. Int. Immunopharmacol..

[42] Tsuchiya S., Kobayashi Y., Goto Y., Okumura H., Nakae S., Konno T. (1982). Induction of maturation in cultured human monocytic leukemia cells by a phorbol diester. Cancer Res..

[43] Tedesco S., De Majo F., Kim J., Trenti A., Trevisi L., Fadini G.P. (2018). Convenience versus biological significance: are PMA-differentiated THP-1 cells a reliable substitute for blood-derived macrophages when studying in vitro polarization?. Front. Pharmacol..

[44] Maeß M.B., Wittig B., Cignarella A., Lorkowski S. (2014). Reduced PMA enhances the responsiveness of transfected THP-1 macrophages to polarizing stimuli. J. Immunol. Methods.

[45] Quinn C.M., Jessup W., Wong J., Kritharides L., Brown A.J. (2005). Expression and regulation of sterol 27-hydroxylase (CYP27A1) in human macrophages: a role for RXR and PPARgamma ligands. Biochem. J..

[46] Worley J.R., Baugh M.D., Hughes D.A., Edwards D.R., Hogan A., Sampson M.J. (2003). Metalloproteinase expression in PMA-stimulated THP-1 cells. Effects of peroxisome proliferator-activated receptor-gamma (PPAR gamma) agonists and 9-cis-retinoic acid. J. Biol. Chem..

[47] Whitney K.D., Watson M.A., Goodwin B., Galardi C.M., Maglich J.M., Wilson J.G. (2001). Liver X receptor (LXR) regulation of the LXRalpha gene in human macrophages. J. Biol. Chem..

[48] Tuoresmäki P., Väisänen S., Neme A., Heikkinen S., Carlberg C. (2014). Patterns of genome-wide VDR locations. PLoS one.

[49] Trinh T.A., Hoang T.X., Kim J.Y. (2020). All-trans retinoic acid increases NF-κB activity in PMA-stimulated THP-1 cells upon unmethylated CpG challenge by enhancing cell surface TLR9 expression. Mol. Cell. Biochem..

[50] Rollins D.A., Kharlyngdoh J.B., Coppo M., Tharmalingam B., Mimouna S., Guo Z. (2017). Glucocorticoid-induced phosphorylation by CDK9 modulates the coactivator functions of transcriptional cofactor GRIP1 in macrophages. Nat. Commun..

[51] Cioni B., Zaalberg A., van Beijnum J.R., Melis M.H.M., van Burgsteden J., Muraro M.J. (2020). Androgen receptor signalling in macrophages promotes TREM-1-mediated prostate cancer cell line migration and invasion. Nat. Commun..

[52] De Bosscher K., Desmet S.J., Clarisse D., Estébanez-Perpiña E., Brunsveld L. (2020). Nuclear receptor crosstalk - defining the mechanisms for therapeutic innovation. Nat. Rev. Endocrinol..

[53] Jin P., Duan X., Huang Z., Dong Y., Zhu J., Guo H. (2025). Nuclear receptors in health and disease: signaling pathways, biological functions and pharmaceutical interventions. Signal Transduct. Targeted Ther..

[54] Yoshikawa T., Ide T., Shimano H., Yahagi N., Amemiya-Kudo M., Matsuzaka T. (2003). Cross-talk between peroxisome proliferator-activated receptor (PPAR) alpha and liver X receptor (LXR) in nutritional regulation of fatty acid metabolism. I. PPARs suppress sterol regulatory element binding protein-1c promoter through inhibition of LXR signaling. Mol. Endocrinol..

[55] Ide T., Shimano H., Yoshikawa T., Yahagi N., Amemiya-Kudo M., Matsuzaka T. (2003). Cross-talk between peroxisome proliferator-activated receptor (PPAR) alpha and liver X receptor (LXR) in nutritional regulation of fatty acid metabolism. II. LXRs suppress lipid degradation gene promoters through inhibition of PPAR signaling. Mol. Endocrinol..

[56] Matsusue K., Miyoshi A., Yamano S., Gonzalez F.J. (2006). Ligand-activated PPARbeta efficiently represses the induction of LXR-dependent promoter activity through competition with RXR. Mol. Cell. Endocrinol..

[57] Juge-Aubry C.E., Gorla-Bajszczak A., Pernin A., Lemberger T., Wahli W., Burger A.G. (1995). Peroxisome proliferator-activated receptor mediates cross-talk with thyroid hormone receptor by competition for retinoid X receptor. Possible role of a leucine zipper-like heptad repeat. J. Biol. Chem..

[58] Alimirah F., Peng X., Yuan L., Mehta R.R., von Knethen A., Choubey D. (2012). Crosstalk between the peroxisome proliferator-activated receptor γ (PPARγ) and the vitamin D receptor (VDR) in human breast cancer cells: pparγ binds to VDR and inhibits 1α,25-dihydroxyvitamin D3 mediated transactivation. Exp. Cell Res..

[59] Wu D.Y., Bittencourt D., Stallcup M.R., Siegmund K.D. (2015). Identifying differential transcription factor binding in ChIP-seq. Front. Genet..

[60] Meyer C.A., Liu X.S. (2014). Identifying and mitigating bias in next-generation sequencing methods for chromatin biology. Nat. Rev. Genet..

[61] Bojcsuk D., Nagy G., Balint B.L. (2017). Inducible super-enhancers are organized based on canonical signal-specific transcription factor binding elements. Nucleic Acids Res..

[62] Cheng C., Gerstein M. (2012). Modeling the relative relationship of transcription factor binding and histone modifications to gene expression levels in mouse embryonic stem cells. Nucleic Acids Res..

[63] Moore D.D., Kato S., Xie W., Mangelsdorf D.J., Schmidt D.R., Xiao R. (2006). International union of pharmacology. LXII. The NR1H and NR1I receptors: constitutive androstane receptor, pregnene X receptor, farnesoid X receptor alpha, farnesoid X receptor beta, liver X receptor alpha, liver X receptor beta, and vitamin D receptor. Pharmacol. Rev..

[64] Mianesaz H., Göczi L., Nagy G., Póliska S., Fadel L., Bojcsuk D. (2025). Genomic regions occupied by both RARα and VDR are involved in the convergence and cooperation of retinoid and vitamin D signaling pathways. Nucleic Acids Res..

[65] Meyer M.B., Benkusky N.A., Lee C.H., Pike J.W. (2014). Genomic determinants of gene regulation by 1,25-dihydroxyvitamin D3 during osteoblast-lineage cell differentiation. J. Biol. Chem..

[66] Meyer M.B., Benkusky N.A., Lee S.M., Yoon S.H., Mannstadt M., Wein M.N. (2022). Rapid genomic changes by mineralotropic hormones and kinase SIK inhibition drive coordinated renal Cyp27b1 and Cyp24a1 expression via CREB modules. J. Biol. Chem..

[67] St John H.C., Bishop K.A., Meyer M.B., Benkusky N.A., Leng N., Kendziorski C. (2014). The osteoblast to osteocyte transition: epigenetic changes and response to the vitamin D3 hormone. Mol. Endocrinol..

[68] Meyer M.B., Goetsch P.D., Pike J.W. (2012). VDR/RXR and TCF4/β-catenin cistromes in colonic cells of colorectal tumor origin: impact on c-FOS and c-MYC gene expression. Mol. Endocrinol..

[69] Martens J.H., Brinkman A.B., Simmer F., Francoijs K.J., Nebbioso A., Ferrara F. (2010). PML-RARalpha/RXR alters the epigenetic landscape in acute promyelocytic leukemia. Cancer Cell.

[70] Tang D., Zhang Z., Zboril E., Wetzel M.D., Xu X., Zhang W. (2021). Pontin functions as A transcriptional Co-activator for retinoic acid-induced HOX gene expression. J. Mol. Biol..

[71] Oda Y., Wong C.T., Oh D.H., Meyer M.B., Pike J.W., Bikle D.D. (2023). Vitamin D receptor cross-talk with p63 signaling promotes epidermal cell fate. J. Steroid Biochem. Mol. Biol..

[72] McCray T., Pacheco J.V., Loitz C.C., Garcia J., Baumann B., Schlicht M.J. (2021). Vitamin D sufficiency enhances differentiation of patient-derived prostate epithelial organoids. iScience.

[73] Lee S.M., Riley E.M., Meyer M.B., Benkusky N.A., Plum L.A., DeLuca H.F. (2015). 1,25-Dihydroxyvitamin D3 controls a cohort of vitamin D receptor target genes in the proximal intestine that is enriched for calcium-regulating components. J. Biol. Chem..

[74] Ratman D., Mylka V., Bougarne N., Pawlak M., Caron S., Hennuyer N. (2016). Chromatin recruitment of activated AMPK drives fasting response genes co-controlled by GR and PPARα. Nucleic Acids Res..

[75] Haakonsson A.K., Stahl Madsen M., Nielsen R., Sandelin A., Mandrup S. (2013). Acute genome-wide effects of rosiglitazone on PPARγ transcriptional networks in adipocytes. Mol. Endocrinol..

[76] Oishi Y., Spann N.J., Link V.M., Muse E.D., Strid T., Edillor C. (2017). SREBP1 contributes to resolution of pro-inflammatory TLR4 signaling by reprogramming fatty acid metabolism. Cell Metab..

[77] Dong L., Zhang D., Cai Y., Zeng M., Mu M., Zhao P. (2025). RACK7 senses and fine-tunes enhancer activity. iScience.

[78] Guo C., Meza-Sosa K.F., Valle-Garcia D., Zhao G., Gao K., Yu L. (2023). The SET oncoprotein promotes estrogen-induced transcription by facilitating establishment of active chromatin. Proc. Natl. Acad. Sci. U. S. A..

[79] Jehanno C., Le Goff P., Habauzit D., Le Page Y., Lecomte S., Lecluze E. (2022). Hypoxia and ERα transcriptional crosstalk is associated with endocrine resistance in breast cancer. Cancers.

[80] Sun J., Gaidosh G., Xu Y., Mookhtiar A., Man N., Cingaram P.R. (2021). RAC1 plays an essential role in estrogen receptor alpha function in breast cancer cells. Oncogene.

[81] Chi D., Singhal H., Li L., Xiao T., Liu W., Pun M. (2019). Estrogen receptor signaling is reprogrammed during breast tumorigenesis. Proc. Natl. Acad. Sci. U. S. A..

[82] Nagarajan S., Rao S.V., Sutton J., Cheeseman D., Dunn S., Papachristou E.K. (2020). ARID1A influences HDAC1/BRD4 activity, intrinsic proliferative capacity and breast cancer treatment response. Nat. Genet..

[83] Holding A.N., Cullen A.E., Markowetz F. (2018). Genome-wide estrogen Receptor-α activation is sustained, not cyclical. eLife.

[84] Helzer K.T., Szatkowski O.M., Meyer M.B., Benkusky N.A., Solodin N., Reese R.M. (2019). The phosphorylated estrogen receptor α (ER) cistrome identifies a subset of active enhancers enriched for direct ER-DNA binding and the transcription factor GRHL2. Mol. Cell Biol..

[85] Reese J.M., Bruinsma E.S., Nelson A.W., Chernukhin I., Carroll J.S., Li Y. (2018). ERβ-mediated induction of cystatins results in suppression of TGFβ signaling and inhibition of triple-negative breast cancer metastasis. Proc. Natl. Acad. Sci. U. S. A..

[86] Guertin M.J., Cullen A.E., Markowetz F., Holding A.N. (2018). Parallel factor ChIP provides essential internal control for quantitative differential ChIP-seq. Nucleic Acids Res..

[87] Mayayo-Peralta I., Gregoricchio S., Schuurman K., Yavuz S., Zaalberg A., Kojic A. (2023). PAXIP1 and STAG2 converge to maintain 3D genome architecture and facilitate promoter/enhancer contacts to enable stress hormone-dependent transcription. Nucleic Acids Res..

[88] Paakinaho V., Lempiäinen J.K., Sigismondo G., Niskanen E.A., Malinen M., Jääskeläinen T. (2021). SUMOylation regulates the protein network and chromatin accessibility at glucocorticoid receptor-binding sites. Nucleic Acids Res..

[89] Hu W., Jiang C., Kim M., Yang W., Zhu K., Guan D. (2021). Individual-specific functional epigenomics reveals genetic determinants of adverse metabolic effects of glucocorticoids. Cell Metab..

[90] Prekovic S., Chalkiadakis T., Roest M., Roden D., Lutz C., Schuurman K. (2023). Luminal breast cancer identity is determined by loss of glucocorticoid receptor activity. EMBO Mol. Med..

[91] Enuka Y., Feldman M.E., Chowdhury A., Srivastava S., Lindzen M., Sas-Chen A. (2017). Epigenetic mechanisms underlie the crosstalk between growth factors and a steroid hormone. Nucleic Acids Res..

[92] Hauck A.K., Mehmood R., Carpenter B.J., Frankfurter M.T., Tackenberg M.C., Inoue S.I. (2024). Nuclear receptor corepressors non-canonically drive glucocorticoid receptor-dependent activation of hepatic gluconeogenesis. Nat. Metab..

[93] Vanderhaeghen T., Timmermans S., Watts D., Paakinaho V., Eggermont M., Vandewalle J. (2022). Reprogramming of glucocorticoid receptor function by hypoxia. EMBO Rep..

[94] Vandewalle J., Timmermans S., Paakinaho V., Vancraeynest L., Dewyse L., Vanderhaeghen T. (2021). Combined glucocorticoid resistance and hyperlactatemia contributes to lethal shock in sepsis. Cell Metab..

[95] Sacta M.A., Tharmalingam B., Coppo M., Rollins D.A., Deochand D.K., Benjamin B. (2018). Gene-specific mechanisms direct glucocorticoid-receptor-driven repression of inflammatory response genes in macrophages. eLife.

[96] Phoenix J.T., Budreika A., Schmeck D.A., Kostlan R.J., Ferrari M.G., Young K.S. (2025). SOX2 utilizes FOXA1 as a heteromeric transcriptional partner to drive proliferation in therapy-resistant prostate cancer. biorxiv.

[97] Eickhoff N., Janetzko J., Padrão N., Gregoricchio S., Siefert J.C., Hoekman L. (2025). TRIM33 loss reduces androgen receptor transcriptional output and H2BK120 ubiquitination. Commun. Biol..

[98] Safi R., Wardell S.E., Watkinson P., Qin X., Lee M., Park S. (2024). Androgen receptor monomers and dimers regulate opposing biological processes in prostate cancer cells. Nat. Commun..

[99] Habault J., Schneider J.A., Ha S., Ruoff R., Pereira L.D., Puccini J. (2023). A multivalent peptoid conjugate modulates androgen receptor transcriptional activity to inhibit therapy-resistant prostate cancer. Mol. Cancer Ther..

[100] Han W., Liu M., Han D., Toure A.A., Li M., Besschetnova A. (2022). Exploiting the tumor-suppressive activity of the androgen receptor by CDK4/6 inhibition in castration-resistant prostate cancer. Mol. Ther..

[101] Chen M., Lingadahalli S., Narwade N., Lei K.M.K., Liu S., Zhao Z. (2022). TRIM33 drives prostate tumor growth by stabilizing androgen receptor from Skp2-mediated degradation. EMBO Rep..

[102] Vélot L., Lessard F., Bérubé-Simard F.A., Tav C., Neveu B., Teyssier V. (2021). Proximity-dependent mapping of the androgen receptor identifies kruppel-like factor 4 as a functional partner. Mol. Cell Proteomics.

[103] Weber H., Ruoff R., Garabedian M.J. (2021). MED19 alters AR occupancy and gene expression in prostate cancer cells, driving MAOA expression and growth under low androgen. PLoS Genet..

[104] Launonen K.M., Paakinaho V., Sigismondo G., Malinen M., Sironen R., Hartikainen J.M. (2021). Chromatin-directed proteomics-identified network of endogenous androgen receptor in prostate cancer cells. Oncogene.

[105] Baumgart S.J., Nevedomskaya E., Lesche R., Newman R., Mumberg D., Haendler B. (2020). Darolutamide antagonizes androgen signaling by blocking enhancer and super-enhancer activation. Mol. Oncol..

[106] Mohammed H., Russell I.A., Stark R., Rueda O.M., Hickey T.E., Tarulli G.A. (2015). Progesterone receptor modulates ERα action in breast cancer. Nature.

[107] Ogara M.F., Rodríguez-Seguí S.A., Marini M., Nacht A.S., Stortz M., Levi V. (2019). The glucocorticoid receptor interferes with progesterone receptor-dependent genomic regulation in breast cancer cells. Nucleic Acids Res..

[108] Nacht A.S., Ferrari R., Zaurin R., Scabia V., Carbonell-Caballero J., Le Dily F. (2019). C/EBPα mediates the growth inhibitory effect of progestins on breast cancer cells. EMBO J..

[109] Davaadelger B., Murphy A.R., Clare S.E., Lee O., Khan S.A., Kim J.J. (2018). Mechanism of telapristone acetate (CDB4124) on progesterone receptor action in breast cancer cells. Endocrinology.

[110] Rubel C.A., Lanz R.B., Kommagani R., Franco H.L., Lydon J.P., DeMayo F.J. (2012). Research resource: genome-wide profiling of progesterone receptor binding in the mouse uterus. Mol. Endocrinol..

[111] Zhan L., Liu H.X., Fang Y., Kong B., He Y., Zhong X.B. (2014). Genome-wide binding and transcriptome analysis of human farnesoid X receptor in primary human hepatocytes. PLoS one.

[112] Lee J., Seok S., Yu P., Kim K., Smith Z., Rivas-Astroza M. (2012). Genomic analysis of hepatic farnesoid X receptor binding sites reveals altered binding in obesity and direct gene repression by farnesoid X receptor in mice. Hepatology.

[113] Su X., Zhang M., Qi H., Gao Y., Yang Y., Yun H. (2022). Gut microbiota-derived metabolite 3-idoleacetic acid together with LPS induces IL-35(+) B cell generation. Microbiome.

[114] Uhlén M., Fagerberg L., Hallström B.M., Lindskog C., Oksvold P., Mardinoglu A. (2015). Proteomics. Tissue-based map of the human proteome. Science.

[115] Töröcsik D., Baráth M., Benko S., Széles L., Dezso B., Póliska S. (2010). Activation of liver X receptor sensitizes human dendritic cells to inflammatory stimuli. J. Immunol..

[116] DeFesi C.R., Fels E.C., Surks M.I. (1985). L-Triiodothyronine (T3) stimulates growth of cultured GC cells by action early in the G1 period: evidence for mediation by the nuclear T3 receptor. Endocrinology.

[117] Kim S., Wysocka J. (2023). Deciphering the multi-scale, quantitative cis-regulatory code. Mol. Cell.

[118] Gasperini M., Hill A.J., McFaline-Figueroa J.L., Martin B., Kim S., Zhang M.D. (2019). A genome-wide framework for mapping gene regulation via cellular genetic screens. Cell.

[119] Simandi Z., Horvath A., Cuaranta-Monroy I., Sauer S., Deleuze J.F., Nagy L. (2018). RXR heterodimers orchestrate transcriptional control of neurogenesis and cell fate specification. Mol. Cell. Endocrinol..

[120] Rampersaud A., Lodato N.J., Shin A., Waxman D.J. (2019). Widespread epigenetic changes to the enhancer landscape of mouse liver induced by a specific xenobiotic agonist ligand of the nuclear receptor CAR. Toxicol. Sci..

[121] Grøntved L., Waterfall J.J., Kim D.W., Baek S., Sung M.H., Zhao L. (2015). Transcriptional activation by the thyroid hormone receptor through ligand-dependent receptor recruitment and chromatin remodelling. Nat. Commun..

[122] Simandi Z., Horvath A., Wright L.C., Cuaranta-Monroy I., De Luca I., Karolyi K. (2016). OCT4 acts as an integrator of pluripotency and signal-induced differentiation. Mol. Cell.

[123] Boergesen M., Pedersen T., Gross B., van Heeringen S.J., Hagenbeek D., Bindesbøll C. (2012). Genome-wide profiling of liver X receptor, retinoid X receptor, and peroxisome proliferator-activated receptor α in mouse liver reveals extensive sharing of binding sites. Mol. Cell Biol..

[124] Cao X., Wang J., Zhang T., Liu Z., Liu L., Chen Y. (2022). Chromatin accessibility dynamics dictate renal tubular epithelial cell response to injury. Nat. Commun..

[125] Loo S.Y., Toh L.P., Xie W.H., Pathak E., Tan W., Ma S. (2021). Fatty acid oxidation is a druggable gateway regulating cellular plasticity for driving metastasis in breast cancer. Sci. Adv..

[126] Pott S., Kamrani N.K., Bourque G., Pettersson S., Liu E.T. (2012). PPARG binding landscapes in macrophages suggest a genome-wide contribution of PU.1 to divergent PPARG binding in human and mouse. PLoS one.

[127] Kulkarni S., Huang J., Tycksen E., Cliften P.F., Rudnick D.A. (2020). Diet modifies pioglitazone's influence on hepatic PPARγ-Regulated mitochondrial gene expression. PPAR Res..

[128] Decker B., Liput M., Abdellatif H., Yergeau D., Bae Y., Jornet J.M. (2020). Global genome conformational programming during neuronal development is associated with CTCF and nuclear FGFR1-The genome archipelago model. Int. J. Mol. Sci..

[129] Yang Y.Z., Subauste J.S., Koenig R.J. (1995). Retinoid X receptor alpha binds with the highest affinity to an imperfect direct repeat response element. Endocrinology.

[130] Dowhan D.H., Downes M., Sturm R.A., Muscat G.E. (1994). Identification of deoxyribonucleic acid sequences that bind retinoid-X receptor-gamma with high affinity. Endocrinology.

[131] Chen Z.P., Iyer J., Bourguet W., Held P., Mioskowski C., Lebeau L. (1998). Ligand- and DNA-induced dissociation of RXR tetramers. J. Mol. Biol..

[132] Penvose A., Keenan J.L., Bray D., Ramlall V., Siggers T. (2019). Comprehensive study of nuclear receptor DNA binding provides a revised framework for understanding receptor specificity. Nat. Commun..

[133] Gampe R.T., Montana V.G., Lambert M.H., Wisely G.B., Milburn M.V., Xu H.E. (2000). Structural basis for autorepression of retinoid X receptor by tetramer formation and the AF-2 helix. Genes Dev..

[134] Zhang Y., Zolfaghari R., Ross A.C. (2010). Multiple retinoic acid response elements cooperate to enhance the inducibility of CYP26A1 gene expression in liver. Gene.

[135] Dilworth F.J., Fromental-Ramain C., Remboutsika E., Benecke A., Chambon P. (1999). Ligand-dependent activation of transcription in vitro by retinoic acid receptor alpha/retinoid X receptor alpha heterodimers that mimics transactivation by retinoids in vivo. Proc. Natl. Acad. Sci. U. S. A..

[136] Bastie J.N., Balitrand N., Guidez F., Guillemot I., Larghero J., Calabresse C. (2004). 1 alpha,25-dihydroxyvitamin D3 transrepresses retinoic acid transcriptional activity via vitamin D receptor in myeloid cells. Mol. Endocrinol..

[137] de Thé H., Vivanco-Ruiz M.M., Tiollais P., Stunnenberg H., Dejean A. (1990). Identification of a retinoic acid responsive element in the retinoic acid receptor beta gene. Nature.

[138] Shulman A.I., Larson C., Mangelsdorf D.J., Ranganathan R. (2004). Structural determinants of allosteric ligand activation in RXR heterodimers. Cell.

[139] Cheskis B., Freedman L.P. (1996). Modulation of nuclear receptor interactions by ligands: kinetic analysis using surface plasmon resonance. Biochemistry.

[140] Prüfer K., Racz A., Lin G.C., Barsony J. (2000). Dimerization with retinoid X receptors promotes nuclear localization and subnuclear targeting of vitamin D receptors. J. Biol. Chem..

[141] Siersbæk M.S., Loft A., Aagaard M.M., Nielsen R., Schmidt S.F., Petrovic N. (2012). Genome-wide profiling of peroxisome proliferator-activated receptor γ in primary epididymal, inguinal, and brown adipocytes reveals depot-selective binding correlated with gene expression. Mol. Cell. Biol..

[142] Barish G.D., Yu R.T., Karunasiri M., Ocampo C.B., Dixon J., Benner C. (2010). Bcl-6 and NF-kappaB cistromes mediate opposing regulation of the innate immune response. Genes Dev..

[143] Csumita M., Csermely A., Horvath A., Nagy G., Monori F., Göczi L. (2020). Specific enhancer selection by IRF3, IRF5 and IRF9 is determined by ISRE half-sites, 5' and 3' flanking bases, collaborating transcription factors and the chromatin environment in a combinatorial fashion. Nucleic Acids Res..

[144] Barta E. (2011). Command line analysis of ChIP-seq results. EMBnet J..

[145] Zhang Y., Liu T., Meyer C.A., Eeckhoute J., Johnson D.S., Bernstein B.E. (2008). Model-based analysis of ChIP-Seq (MACS). Genome Biol..

[146] ENCODE-Project-Consortium (2012). An integrated encyclopedia of DNA elements in the human genome. Nature.

[147] Thorvaldsdóttir H., Robinson J.T., Mesirov J.P. (2013). Integrative genomics viewer (IGV): high-performance genomics data visualization and exploration. Brief. Bioinformatics.

[148] Heinz S., Benner C., Spann N., Bertolino E., Lin Y.C., Laslo P. (2010). Simple combinations of lineage-determining transcription factors prime cis-regulatory elements required for macrophage and B cell identities. Mol. Cel..

[149] Stark R., Brown G. (2011). DiffBind: differential binding analysis of ChIP-Seq peak data. R. Package Version.

