# Supplementary material for: Genome-wide chromatin profiling reveals a nonlimiting role for RXR in macrophage-like cells stimulated with multiple nuclear receptor agonists
Source: J Biol Chem. 2026 Apr 22;302(6):111486. doi: 10.1016/j.jbc.2026.111486 (PMC13241730; doi:10.1016/j.jbc.2026.111486)
Supplement: Supplementary Figures S1 to S7 [file mmc1.pdf]

Supplementary Figure S1

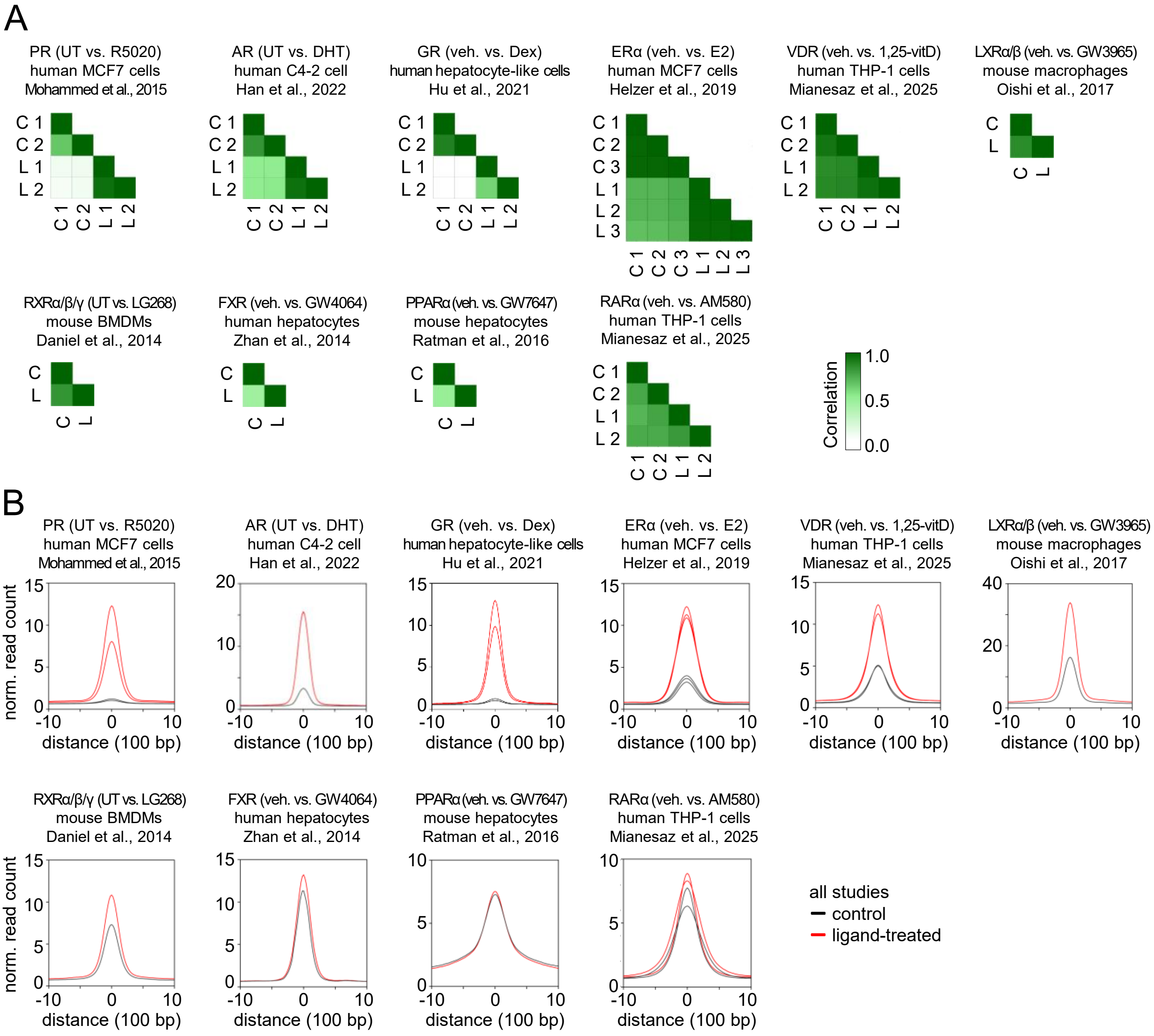

Supplementary Figure S1. Ligand-dependent effects on DNA occupancy of nuclear receptors. (A) Correlation analyses of ChIP-seq peak sets in control and ligand-stimulated cells from representative studies. Correlations were calculated using genome-wide binding signal intensities across samples. (B) Histograms showing normalized read counts within ±1000 bp of ChIP-seq peak summits in control and ligand-stimulated cells from the same representative studies. Control and ligand-treated replicates are shown in black and red, respectively. From the studies available for each receptor (see meta-analysis), a single representative study was chosen for panels A and B on the basis of having a ligand-to-control global occupancy ratio close to the median value calculated across all studies for that receptor. C, control; L, ligand; veh., vehicle; UT, untreated.

Supplementary Figure S2

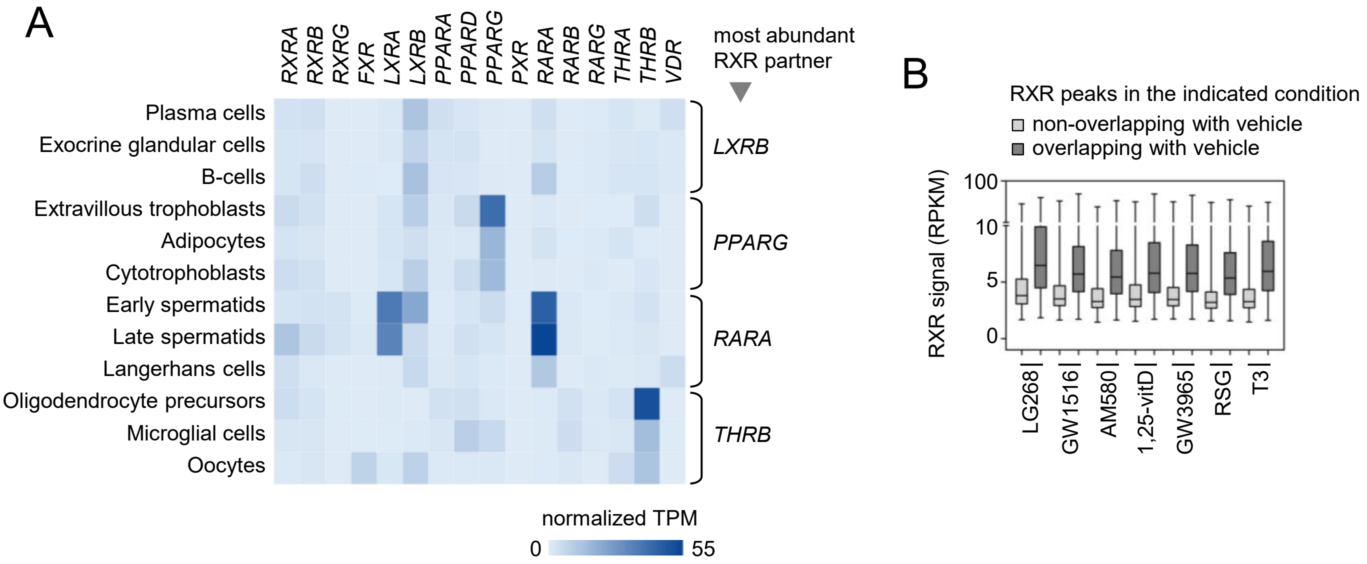

Supplementary Figure S2. Expression of RXRs and their heterodimerization partners across various cell types, and RXR occupancy in PMA-THP1 cells under different ligand treatments. (A) Heatmap showing the expression levels of RXR isoforms and their ligand-inducible partners across representative cell types. RNA-seq data were obtained from the Human Protein Atlas. (B) Box plot showing RXR occupancy values within RXR binding regions measured under different ligand treatments. RXR binding regions were grouped based on their overlap with RXR peaks identified in vehicle-treated cells.

Supplementary Figure S3

A

ligand	RXR peaks FC > 1.5, FDR < 0.05	RXR peaks FC < 0.66, FDR < 0.05
LG268	8820 (11.8%)	10430 (14.0%)
GW1516	0 (0.0%)	0 (0.0%)
AM580	325 (0.5%)	0 (0.0%)
1,25-vitD	802 (1.3%)	55 (0.1%)
GW3965	9 (0.0%)	0 (0.0%)
RSG	633 (0.9%)	56 (0.1%)
T3	1490 (1.9%)	726 (0.9%)

B

ligand	RXR peaks FC > 1.5	RXR peaks FC < 0.66
LG268	13399 (18.0%)	14929 (20.1%)
GW1516	1737 (2.8%)	1645 (2.6%)
AM580	2127 (3.3%)	1227 (1.9%)
1,25-vitD	2390 (3.8%)	1987 (3.2%)
GW3965	2934 (4.5%)	1671 (2.5%)
RSG	6789 (10.0%)	2789 (4.1%)
T3	8606 (11.2%)	6566 (8.5%)

C

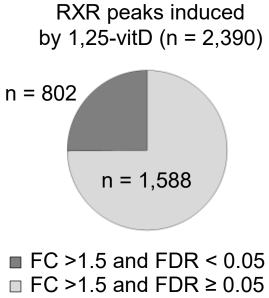

D

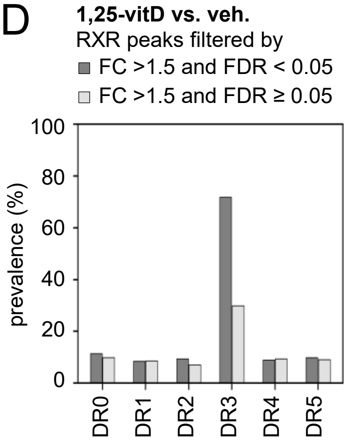

E

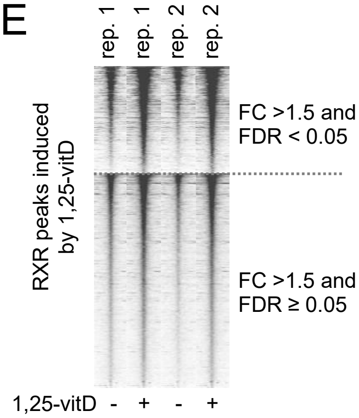

F

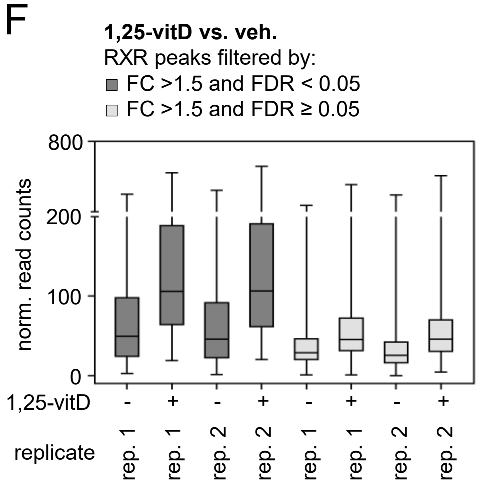

G

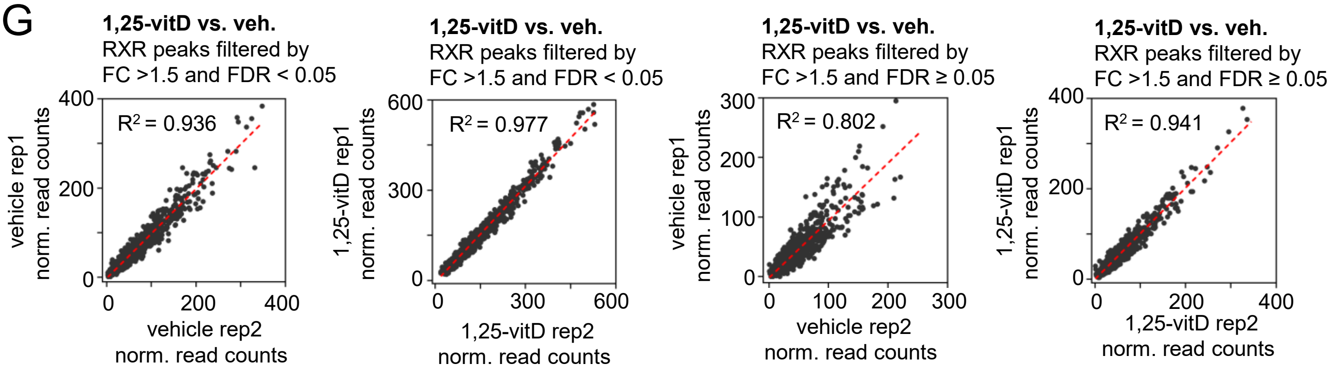

Supplementary Figure S3. Identification of RXR binding regions showing changes in RXR occupancy after ligand treatment using different filtering strategies. (A-B) Tables summarizing the number of RXR binding regions with changes in RXR occupancy upon ligand treatment. For each treatment condition, consensus peak sets were determined, and the fold change (FC) in RXR occupancy between ligand-treated and vehicle-treated samples, as well as the false discovery rate (FDR), were calculated using DiffBind. Peaks were filtered using combined FC and FDR cutoffs (A) or using FC cutoff only to define the exploratory RXR peak sets (B). Percentages in parentheses indicate the fraction of the total number of RXR peaks identified in each treatment condition which passed the indicated filtering criteria. (C) Pie chart showing the proportions of the two subsets of 1,25-vitD-induced RXR peaks, defined using filters based on FC with or without FDR filtering. (D) Prevalence of DR0-DR5 motifs in the two different subsets of 1,25-vitD-induced RXR peaks. (E) RD plots displaying ChIP-seq signals for the two subsets of 1,25-vitD-induced RXR peaks for both replicates of vehicle- and 1,25-vitD-treated cells within a ±2 kb window centered on peak summits. (F) Box plots showing normalized read counts for both replicates in both conditions for the two subsets of 1,25-vitD-induced RXR peaks. (G) Scatter plots showing the correlation of normalized RXR signals between the two biological replicates for each condition (vehicle or 1,25-vitD) and for the two subsets of 1,25-vitD-induced RXR peaks, defined using FC and FDR filtering criteria.

Supplementary Figure S4

A

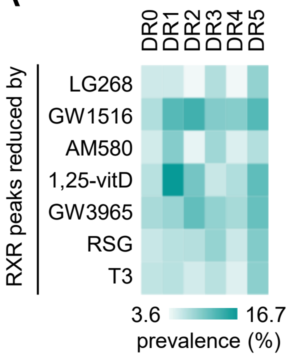

B

RXR dimer	canonical RE
RXR-RXR	DR1
PPAR-RXR	DR1
RAR-RXR	DR1, DR2 and DR5
VDR-RXR	DR3
TR-RXR	DR4
LXR-RXR	DR4

C

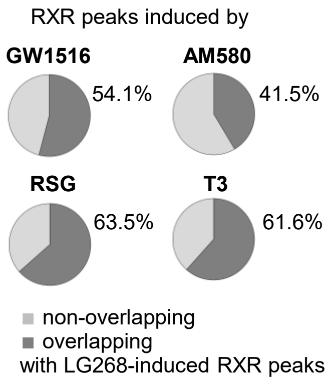

Supplementary Figure S4. Characterization of exploratory RXR peak sets showing changes in RXR occupancy after ligand treatment. (A) Frequency of the indicated DNA motifs detected within exploratory RXR peak sets reduced by ligand treatment. (B) Canonical response elements (RE) preferred by RXR homo- and heterodimers examined in this study, as reported in the literature. DR, direct repeat. (C) Proportion of RXR peaks induced by each ligand that overlap with LG268-induced RXR peaks.

Supplementary Figure S5

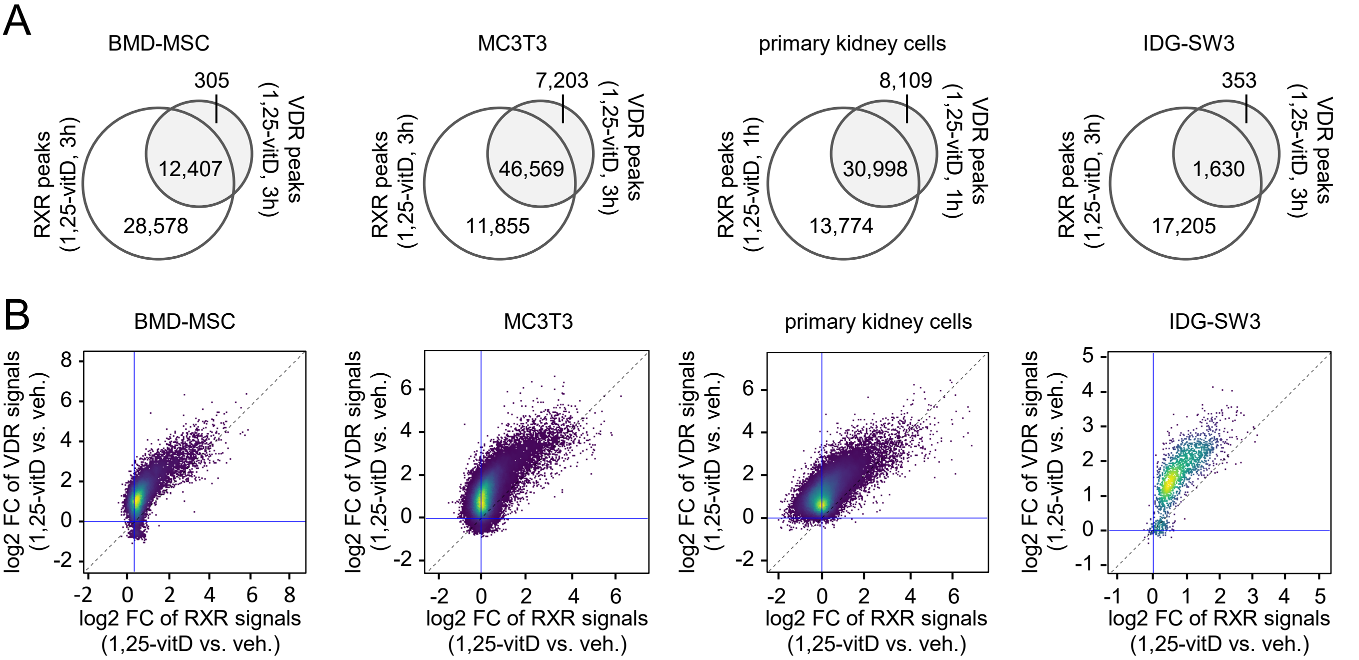

Supplementary Figure S5. Changes in RXR and VDR occupancy upon 1,25-vitD treatment in four studies. (A) Venn diagrams illustrating the overlap of RXR and VDR ChIP-seq peaks in cells treated with 1,25-vitD across four murine cell types. (B) Pseudocolor scatter plots showing 1,25-vitD–induced changes in RXR and VDR binding in the same cell types. At common RXR and VDR binding regions, occupancy signals were calculated for both RXR and VDR in vehicle- and 1,25-vitD–treated cells. Fold changes (FC) between 1,25-vitD and vehicle were calculated for RXR and VDR and plotted against each other. RXR and VDR ChIP-seq datasets were obtained from the following studies: Bone Marrow-Derived Mesenchymal Stem Cells (BMD-MSC; Meyer et al., 2016); osteoblast precursor cell line (MC3T3; Meyer et al., 2014); primary kidney cells (Meyer et al., 2022); and osteocytogenic cell line (IDG-SW3; St John et al., 2014).

Supplementary Figure S6

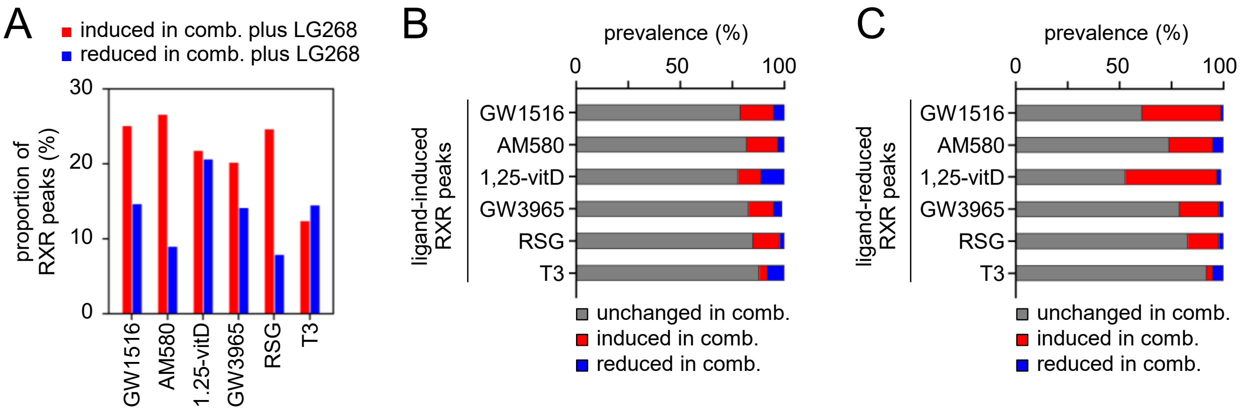

Supplementary Figure S6. Effect of ligand co-treatment on RXR occupancy relative to single-ligand treatments. (A) Proportions of RXR peaks that were induced (RXR signal ratio > 1.5) or reduced (RXR signal ratio < 0.66) in cells treated with six agonists for RXR partners together with LG268 (combined plus LG268), relative to the corresponding single-ligand treatments. (B–C) Proportions of ligand-induced (B) and ligand-reduced (C) exploratory RXR peak sets that were unchanged and further induced or reduced (cut-offs: >1.5 and <0.667) in cells treated with combined treatment, relative to the corresponding single-ligand treatments.

Supplementary Figure S7

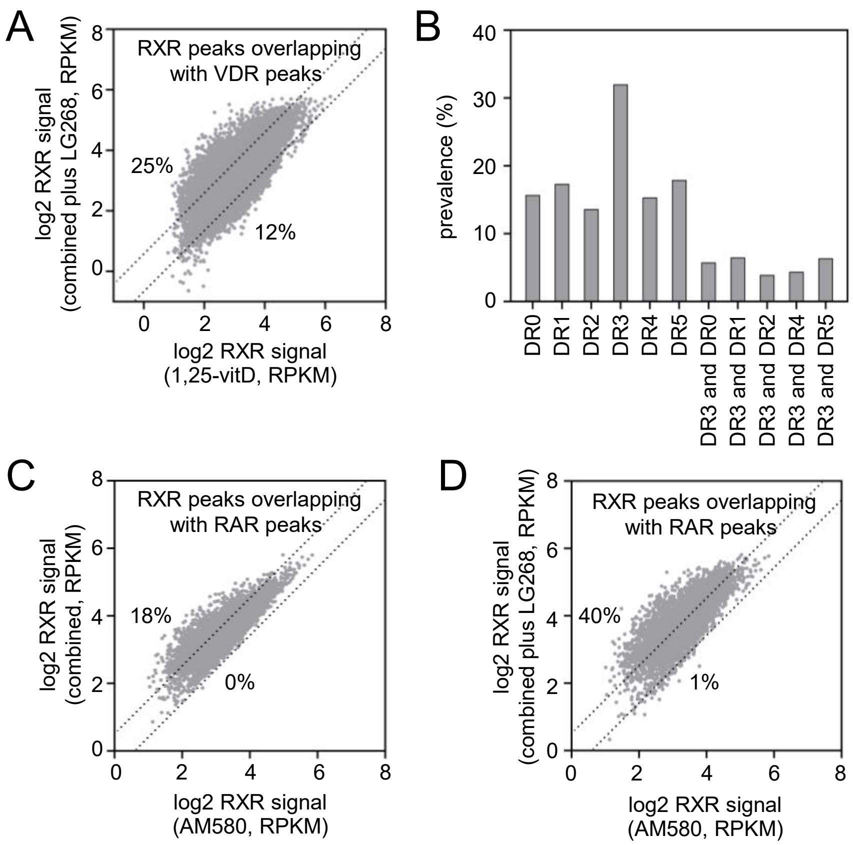

Supplementary Figure S7. Comparison of ligand co-treatment with single ligand treatments (1,25-vitD or AM580) on RXR occupancy. (A) Scatter plot showing RXR signal intensities in cells treated with six agonists for RXR partners together with LG268 (combined plus LG268), compared with cells treated with the VDR agonist (1,25-vitD). RXR peaks overlapping with VDR peaks are displayed. Dotted lines indicate RXR signal ratios of 1.5 and 0.66. The proportion of RXR peaks above or below these thresholds is shown. (B) Prevalence of different motifs in RXR peaks overlapping with VDR peaks. (C-D) For the indicated comparisons, scatter plots show RXR signal intensities in cells treated with either six agonists for RXR partners (combined) (C) or the same six agonists together with LG268 (combined plus LG268) (D), compared with cells treated with the RAR agonist (AM580) alone. RXR peaks overlapping with RAR peaks are displayed. Dotted lines indicate RXR signal ratios of 1.5 and 0.66. The proportion of RXR peaks above or below these thresholds is shown.
