# Supplementary material for: Genome-wide chromatin profiling reveals a nonlimiting role for RXR in macrophage-like cells stimulated with multiple nuclear receptor agonists
Source: J Biol Chem. 2026 Apr 22;302(6):111486. doi: 10.1016/j.jbc.2026.111486 (PMC13241730; doi:10.1016/j.jbc.2026.111486)
Supplement: Supplementary Tables legends [file mmc2.docx]

**Supplementary Table legends**

**Supplementary Table S1.** Studies with nuclear receptor (NR) ChIP-seq datasets that met the selection criteria for meta-analysis of ligand-dependent DNA occupancy.

**Supplementary Table S2.** List of human NRs according to the HUGO Gene Nomenclature Committee (HGNC). The table provides information on RXR dimerization and the identified endogenous ligand(s) according to the literature.

**Supplementary Table S3.** Exploratory RXR peak sets showing changes in RXR occupancy after ligand treatment as determined by ChIP-seq in ligand-treated PMA-THP-1 cells. Consensus peak sets were determined for each ligand-treatment condition, and fold changes (FC) in RXR occupancy (ligand vs. vehicle) and false discovery rate (FDR) were calculated using DiffBind. Peaks passing the fold-change cutoff (FC > 1.5 or FC < 0.66667) were retrieved to generate an exploratory set of RXR peaks with changed RXR occupancy upon treatment with each ligand.

**Supplementary Table S4.** Differentially expressed genes in 1,25-vitD-stimulated PMA-THP-1 cells. Genes were filtered using cut-offs of adjusted p-value < 0.05 and FC > 1.5 or FC < 0.6667 for up- and downregulated genes, respectively.

**Supplementary Table S5.** RXR peak sets located in proximity to 1,25-vitD-induced genes. Consensus RXR ChIP-seq peaks in cells treated with vehicle or 1,25-vitD for 1 hour were determined using DiffBind. Peaks were categorized using cut-offs of FC > 1.5 for “1,25-vitD-induced RXR peaks,” FC < 0.6667 for “1,25-vitD reduced RXR peaks,” and 0.6667 ≤ FC ≤ 1.5 for “1,25-vitD-unresponsive RXR peaks”. Peaks located within TSS ± 25 kb of 1,25-vitD-induced genes were identified, and for each peak, the average ChIP-seq signal from two replicates of the same condition for RXR, VDR, MED1, and H3K27ac under vehicle or 1,25-vitD stimulation, the highest DR3 motif score within the peak region, and the associated 1,25-vitD-induced gene(s) are provided.

**Supplementary Table S6.** mRNA levels and cumulative RXR occupancy near 1,25-vitD-induced genes. Genes with at least one RXR peak within TSS ± 25 kb are included. The table provides mRNA levels and cumulative RXR occupancy at RXR ChIP-seq peaks within TSS ± 25 kb for each gene under vehicle, 1,25-vitD, and combined (six agonists of RXR partners) ligand treatment conditions.
